# PPM-18, an Analog of Vitamin K, Induces Autophagy and Apoptosis in Bladder Cancer Cells Through ROS and AMPK Signaling Pathways

**DOI:** 10.3389/fphar.2021.684915

**Published:** 2021-07-09

**Authors:** Huiai Lu, Chunlei Mei, Luhao Yang, Junyan Zheng, Junwei Tong, Fengsen Duan, Huageng Liang, Ling Hong

**Affiliations:** ^1^ Department of Biology, College of Life Science and Technology, Huazhong University of Science and Technology, Wuhan, China; ^2^ National Engineering Research Center for Nanomedicine, College of Life Science and Technology, Huazhong University of Science and Technology, Wuhan, China; ^3^ Tongji Medical College, Huazhong University of Science and Technology, Wuhan, China; ^4^ Department of Urology, Union Hospital, Tongji Medical College, Huazhong University of Science and Technology, Wuhan, China

**Keywords:** PPM-18, bladder cancer, apoptosis, autophagy, reactive oxygen species, AMP-activated protein kinase

## Abstract

PPM-18, identified as a novel analog of vitamin K, has been reported to play a critical role in the suppression of seizures. However, the concerns that whether PPM-18, like vitamin K, exerts anticancer activity remain to be further investigated. Here, we found that PPM-18 remarkably suppressed the proliferation and induced apoptosis in bladder cancer cells. Furthermore, a significant autophagic effect of PPM-18 on bladder cancer cells was also demonstrated, which profoundly promoted apoptotic cell death. Mechanistically, PPM-18 activated AMP-activated protein kinase (AMPK), whereas it repressed PI3K/AKT and mTORC1 pathways in bladder cancer cells. Inhibition of AMPK markedly relieved PPM-18–induced autophagy and apoptosis, indicating that PPM-18 is able to induce autophagy and apoptosis in bladder cancer cells *via* AMPK activation. Moreover, reactive oxygen species (ROS) were notably accumulated in PPM-18–treated bladder cancer cells, and treatment with ROS scavengers not only eliminated ROS production but also abrogated AMPK activation, which eventually rescued bladder cancer cells from PPM-18–triggered autophagy and apoptotic cell death. In bladder cancer xenografts, the anticancer activities of PPM-18, including suppressing the growth of tumors and inducing autophagy and apoptosis in tumor cells, were also established. Collectively, this study was the first to demonstrate the anticancer effect of PPM-18 on bladder cancer cells *in vitro* and *in vivo* through eliciting autophagy and apoptosis *via* ROS and AMPK pathways, which might provide new insights into the potential utilization of PPM-18 for future bladder cancer treatment.

## Introduction

Bladder cancer is one of the most common and lethal malignant carcinomas in the human urinary system and ranks the 10th of cancer occurrences worldwide. In 2020, approximately 573,000 new bladder cancer cases and 213,000 deaths were documented. Moreover, bladder cancer is the sixth most common cancer and the ninth leading cause of cancer deaths in men, and is a severe threat to human health (Sung et al., 2021). Although traditional therapeutics, such as radical cystectomy, chemotherapy, radiotherapy, and immunotherapy, have currently made substantial progress in bladder cancer treatment (James et al., 2012; Bruins et al., 2014; Trenta et al., 2016; Pettenati and Ingersoll, 2018), the adverse effects, including distant metastasis, local relapse, toxicity to normal tissues, and lower survival rate of patients, remain inevitable (Soloway, 2013; Feuerstein and Goenka, 2015; Salama et al., 2016). Therefore, exploring novel and highly effective drugs against bladder cancer becomes urgent.

PPM-18 (NQN-1) shares a naphthoquinone moiety with the vitamin K family and is identified as a novel analog of vitamin K. Recent studies have revealed that both vitamin K and PPM-18 could repress seizures in zebrafish or mouse epilepsy models by increasing cellular respiration and total ATP production (Rahn et al., 2014). Moreover, another study showed that PPM-18 also belonged to the histone deacetylase (HDAC) inhibitor family and was capable of selectively killing leukemia cells through inhibition of HDAC6, a class IIb of HDACs (Inks et al., 2012). Nevertheless, the abilities of PPM-18 against solid tumors remain unclear. Furthermore, the exact mechanism through which PPM-18 functions as a potential tumor suppressor still needs to be investigated.

AMP-activated protein kinase (AMPK) is a master regulator that maintains intracellular energy homeostasis. In response to energy shortage, AMPK is rapidly activated and promotes catabolic metabolism to generate ATP. Accordingly, anabolic metabolism that consumes ATP is suppressed by activating AMPK (Garcia and Shaw, 2017). In a tumor microenvironment, cancer cells are frequently confronted with energy stress due to their rapid proliferation and limited nutrient supply (Zhao et al., 2017). The activated AMPK is able to rewire metabolism through catabolism promotion for ATP generation and also regulates the intracellular redox state through alleviating NADPH depletion for reactive oxygen species (ROS) removal, which profoundly promotes cancer cell survival (Jeon et al., 2012). However, AMPK is a double-edged sword. In some circumstance, AMPK activation is also proved to act as a tumor suppressor to elicit autophagic or apoptotic cell death in multiple cancer cells in response to a variety of stimuli, such as glucose deprivation, energy shortage, and intracellular oxidative stress (Lu et al., 2019; Sun et al., 2019; Duan et al., 2020). In this regard, AMPK has also been recognized as a barrier for cancer progression. Many studies indicate that the deficiency of AMPK is prevalent in several types of cancer cells, including bladder cancer (Fox et al., 2013; Zheng et al., 2013; Kopsiaftis et al., 2016). Correspondingly, increasing AMPK expression or restoring AMPK activity efficiently hampers the growth of cancer cells through the induction of autophagic or apoptotic cell death. As a result, targeting AMPK seems to be a promising strategy for cancer treatment.

Increased ROS levels have been observed in cancer cells as compared to normal cells. Early studies showed that ROS elevation was able to damage DNA, which promoted tumorigenesis by increasing genomic instability (Milkovic et al., 2019). Current studies also reveal that higher ROS commonly act as tumorigenic regulators to promote cellular proliferation, metabolic alteration, and angiogenesis by mediating several signal pathways, which eventually facilitates cancer progression (Sullivan and Chandel, 2014). However, ROS are double-edged swords as well. Further increasing ROS production that exceeds the lethal threshold is detrimental to cellular structures, such as the lipid membrane, nucleic acid, and protein, which ultimately leads to cancer cell death (Gorrini et al., 2013; Nogueira and Hay, 2013; Fan et al., 2020). Accordingly, cancer cells usually upregulate the expression of antioxidant enzymes to counteract the sustained ROS generation due to the rapid metabolism and proliferation (Cox et al., 2009; Hayes et al., 2020), which protects cancer cells from oxidative stress–induced cell death. Therefore, directly increasing ROS generation or targeting antioxidant enzymes appear to be wise options for cancer treatment.

Given the urgent need for exploring novel drugs against bladder cancer and the uncertain suppressive role of PPM-18 in solid tumors, this study aimed to evaluate the anticancer effect of PPM-18 on bladder cancer cells *in vitro* and *in vivo*. Furthermore, the regulatory roles of ROS and AMPK pathways in autophagy and apoptosis were also investigated, upon PPM-18 treatment, which discloses the underlying anticancer mechanism of PPM-18 in bladder cancer cells.

## Materials and Methods

### Reagents

PPM-18, purity ≥95%, was purchased from Cayman Chemical Company (Ann Arbor, MI, United States). The pan-caspase inhibitor Z-VAD-FMK was purchased from Beyotime (Shanghai, China). N-acetyl-L-cysteine (NAC) and vitamin E were purchased from Sigma (St.Louis, MO, United States). Glutathione (GSH) was purchased from Ke Rui (Wuhan, China). 3-Methyladenine (3-MA), chloroquine (CQ), and rapamycin were purchased from MCE (Shanghai, China). Compound C was purchased from Selleck (Selleck Chemicals, Shanghai, China).

### Cell Culture

Human bladder cancer T24 cell line was purchased from the China Center for Type Culture Collection (Wuhan University Collection Center, Wuhan, China). Human bladder cancer cell line EJ and human bladder immortalized epithelium cell line SV-HUC-1 were kindly provided by Associate Professor Huageng Liang, Department of Urology, Union Hospital of Tongji Medical College, Huazhong University of Science and Technology, China. Human normal liver cell line L02 was purchased from iCell Bioscience Inc. (Shanghai, China). Human embryonic kidney cell line HEK293T was obtained from American Type Culture Collection (ATCC). Human lung cancer A549, cervical cancer Hela, glioma cancer U87, breast cancer MCF-7 and MDA-MB-231, and prostate cancer PC-3 cell lines were obtained from the College of Life Science and Technology, Huazhong University of Science and Technology (Wuhan, China). T24, EJ, A549, MCF-7, and U87 cells were cultured in Eagle’s minimum essential medium (MEM) supplemented with 10% fetal bovine serum (FBS). L02, HEK293T, Hela, MDA-MB-231, and PC-3 cell lines were cultured in Dulbecco’s modified Eagle’s medium (DMEM) supplemented with 10% fetal bovine serum (FBS). SV-HUC-1 cells were grown in Ham’s F-12K medium supplemented with 10% FBS. All the cultures were maintained at 37°C in a humidified 5% CO_2_ incubator.

### Cell Viability Assay

Cells were seeded in 96-well plates at a density of 1 × 10^4^ cells per well. After being cultured for 24 h, the cells were exposed to the indicated concentration of PPM-18 combined with or without the correlative inhibitors. Cell viability was then assessed by the MTS assay following the manufacturer’s protocol (G3580, Promega, United States) and measured using a microplate reader (Bio-Rad, United States) at 490 nm.

### Bromodeoxyuridine ELISA Assay

Cell proliferation was tested using the BrdU cell proliferation assay kit (X1327K1, Exalpha Biologicals, Shirley, MD). In brief, cells were seeded in 96-well plates at a density of 1 × 10^4^ cells per well. After being cultured for 12 h, the cells were exposed to the indicated concentration of PPM-18 with a labeling medium containing BrdU at 37°C and a 5% CO_2_ incubator. Cells were then fixed and processed for BrdU detection according to the manufacturer’s protocol.

### Cellular Colony Formation Assay

Cells were seeded in 12-well plates at a density of 500–1,000 cells each well and cultured for three days. The cells were then cultured in the medium containing the indicated concentration of PPM-18 for 12 h. After removing the medium containing PPM-18, a fresh medium without PPM-18 was added for further culture till visible cellular colony formation. After fixing with anhydrous methanol and washing with PBS three times, cell colonies were subject to 1% crystal violet (Biosharp, China) for staining and then photographed using a camera.

### Apoptosis Analysis

Cells were seeded in 12-well plates at a density of 1 × 10^6^ cells per well. Then, the cells were treated with the indicated concentration of PPM-18 in the presence or absence of the correlative inhibitors. To determine cell apoptosis, cells were trypsinized, washed with PBS, and suspended in 500 μL binding buffer. The cells were then stained with FITC-conjugated annexin V and propidium iodide (PI) for 15 min, in accordance with the manufacturer’s protocol (556547, BD Biosciences, San Jose, CA, United States). The apoptotic cells were analyzed by flow cytometry (CytoFlex, Beckman-Coulter).

### Western Blotting

Cells were seeded in 6-well plates at a density of 1 × 10^6^ cells per well. After being exposed to the indicated concentration of PPM-18 combined with or without the correlated inhibitors or siRNA, cells were lysed with radioimmunoprecipitation (RIPA) buffer (Beyotime, China) supplemented with a protease inhibitor cocktail (Beyotime, China) for 30 min on ice. Total protein samples were obtained after cell lysates were centrifuged at 12,000×g for 20 min. The total protein was then resolved by SDS-PAGE and transferred into PVDF membranes (Millipore, United States). The membranes containing the targeted protein were subsequently blocked with 5.0% fat-free milk for 2 h and probed with primary antibodies overnight at 4°C, then secondary antibodies for 2 h at room temperature. The protein expression was detected by Odyssey_CLx (LI-COR Company, United States). In this study, western blotting was repeated at least three times, and the density of each band was quantified by densitometry using Image Studio software and then normalized to loading controls. The antibodies utilized in western blotting are as follows: anti-cleaved caspase-9 (BA3974, BOSTER), anti-cleaved caspase-3 (9662, Cell Signaling Technology), anti-cleaved PARP (9542, Cell Signaling Technology), anti-BAX (2772, Cell Signaling Technology), anti-BCL-2 (2872, Cell Signaling Technology), anti-p62 (ab109012, Abcam), anti-LC3B (ab192890, Abcam), anti-phospho-AMPKα (Thr172) (2535, Cell Signaling Technology), anti-AMPKα (A5008, Selleck), anti-phospho-mTORC1 (ser2448) (5536, Cell Signaling Technology), anti-phospho-P70S6K (Thr389) (9234, Cell Signaling Technology), anti-phospho-PI3K (abs130868, Absin Bioscience), anti-PI3K (4249, Cell Signaling Technology), anti-phospho-Akt (ser473) (4060, Cell Signaling Technology), anti-AKT (ab188099, Abcam), anti-SOD1 (4266, Cell Signaling Technology), anti-SOD2 (13141, Cell Signaling Technology), anti-Catalase (ab76110, Abcam), anti-β-actin (A01011, Abbkine), anti-GAPDH (A01020, Abbkine), anti-rabbit IgG (H+L) (DyLightTM 800 4X PEG Conjugate) (5151, Cell Signaling Technology), and anti-mouse IgG (H+L) (DyLightTM 800 Conjugate) (5257, Cell Signaling Technology).

### Mitochondrial Membrane Potential Assay

Cells were seeded in 12-well plates at a density of 1 × 10^6^ cells per well and exposed to 10 μM PPM-18 combined with or without 6 mM NAC, 5 mM GSH, 2 mM VE for 24 h. Cells were collected, washed with PBS, and then stained with JC-1 (Yeasen, Shanghai, China) at 37°C for 30 min. The mitochondrial membrane potential was measured by the intensity of JC-1 red fluorescence (high potential) and JC-1 green fluorescence (low potential) by flow cytometry (FC500, Beckman Coulter, United States) with the excitation and emission wavelengths of 530 nm, 590 nm (red), and 485 nm, 528 nm (green), respectively.

### Transmission Electron Microscopy Assay

T24 and EJ cells were seeded in 6-well plates, and treated with or without 10 μM PPM-18 for 18 h, then washed with PBS, trypsinized, and harvested. Cell pellets were fixed with 2.5% glutaraldehyde, and ultrathin sections were prepared. The formation of autophagosomes in bladder cancer cells was observed under the transmission electron microscope (Olympus, Japan).

### Measurement of Intracellular ROS

Cells were seeded in 12-well plates and exposed to the indicated concentration of PPM-18 combined with or without 6 mM NAC, 5 mM GSH, or 2 mM VE for 12 h. To measure the intracellular ROS production, the cells were collected and then stained with 1 μM 2′,7′-dichlorofluorescein-diacetate (DCFH-DA) (Applygen, China) at 37°C for 30 min. Cells were washed three times with PBS and then analyzed by flow cytometry (CytoFlex, Beckman-Coulter).

### Transfection Assay

Cells were seeded in 6-well plates at a density of 1 × 10^5^ cells per well and cultured for 24 h. The cells were then transfected with siRNA using Lipofectamine 2000 (11668019, Invitrogen). After 72 h of interference, the cells were treated with or without 10 μM PPM-18 for 24 h. The siRNA targeting human AMPK in this study was synthesized by the Gene Pharma Company (Suzhou, China). The sequences of siRNA were as follows: siAMPK: 5′-CAA​AUG​CUU​CCA​UUU​GUA​ATT-3′ and siControl: 5′-UUC​UCC​GAA​CGU​GUC​ACG​UTT-3′.

### Tumor Xenograft Assay

Female BALB/c nude mice (4–5 weeks old) were purchased from Beijing Hua Fukang Bioscience Company (Beijing, China) and bred in specific pathogen-free (SPF) conditions at 25°C under a 12 h light-dark cycle with free access to food and water. All animal experiments were approved by the Ethical Committee on Animal Experimentation of Tongji Medical College, Huazhong University of Science and Technology, China. Briefly, the human bladder cancer xenografts were constructed by subcutaneously injecting 1 × 10^7^ EJ cells into the right flanks of BALB/c mice. When the tumor sizes reached approximately 100 mm^3^, the tumor-bearing mice were randomly divided into the normal controls group (*n* = 7) and PPM-18–treated group (*n* = 7). PPM-18 (10 mg/kg) was dissolved in saline and administered each day by intratumoral injection for 30 days, while controls were injected with the equivalent volume of saline. The tumor sizes and weights of the mice were measured every three days. Tumor volume was calculated by the formula: length × width^2^/2. After 30 days of treatment, the mice were sacrificed, and the tumors were excised and fixed for immunohistochemistry (IHC), TUNEL staining and ROS detecting. In addition, several important organs, including the heart, liver, spleen, lung, and kidney, were also fixed in 4% paraformaldehyde (PFA) and embedded in paraffin. The sections were then subjected to hematoxylin and eosin staining (H&E).

### Statistical Analysis

All data were presented as the mean ± SD of at least three times independent experiments and analyzed using the GraphPad Prism 6 software. Differences between groups were measured using Student’s *t* test. The statistical significance in the figures was regarded as *p < 0.05, **p < 0.01, and ***p < 0.001.

## Results

### PPM-18 Suppresses the Proliferation of Bladder Cancer Cells

**FIGURE 1:**
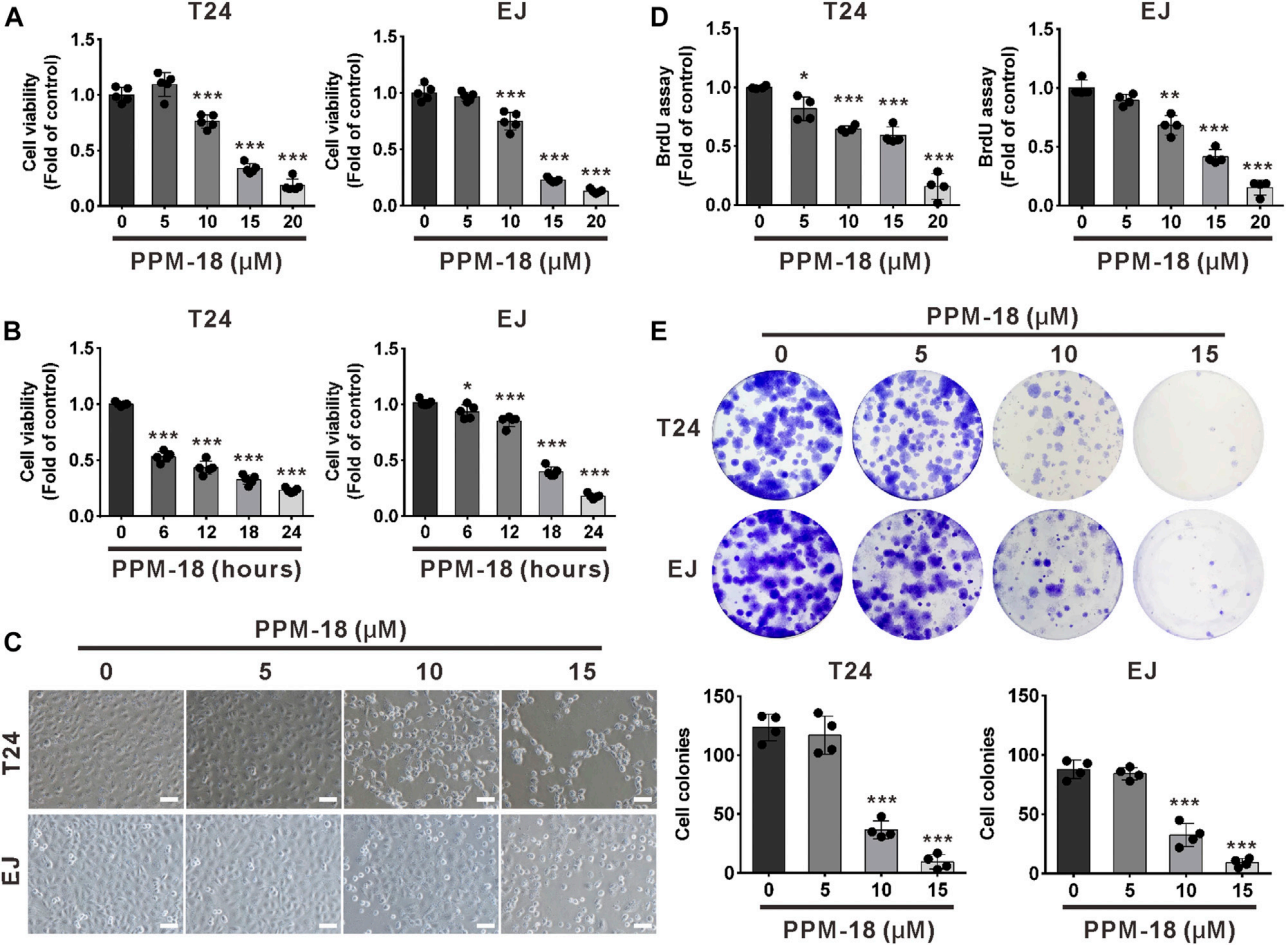
PPM-18 inhibits the growth of bladder cancer cells. **(A)** Bladder cancer T24 and EJ cells were dose-dependently treated with PPM-18 for 24 h, and the cell viability was assessed by the MTS assay. **(B)** The effect of 15 μM PPM-18 on the viability of T24 and EJ cells in a time-dependent manner. **(C)** T24 and EJ cells were treated with the indicated concentration of PPM-18 for 24 h, and the morphological changes of cells were observed under the microscope. Scale bar: 100 µm. (**D)** The effect of PPM-18 on the proliferation of T24 and EJ cells was determined by the BrdU assay. **(E)** PPM-18 dose-dependently affected the cellular colony formation of T24 and EJ cells. Data are presented as the mean ± SD of at least three independent experiments. *p < 0.05, **p < 0.01, and ***p < 0.001 vs. the control group.

To investigate whether PPM-18 inhibits the proliferation of bladder cancer cells, we first assessed the effect of PPM-18 on the viability of bladder cancer cells. As shown in Figures 1A,B, PPM-18 remarkably reduced the viability of bladder cancer T24 and EJ cells in a dose- or time-dependent manner. Moreover, significant morphological alterations were exhibited in PPM-18–treated T24 and EJ cells (Figure 1C). To further determine the inhibitory growth effect of PPM-18 on bladder cancer cells, BrdU and cellular colony assays were performed. As shown in Figure 1D, PPM-18 dose-dependently repressed the BrdU incorporation in T24 and EJ cells. Moreover, cellular colony formation was markedly inhibited following exposure to the increasing concentration of PPM-18 (Figure 1E). These results indicate that PPM-18 is capable of suppressing the proliferation of bladder cancer cells. In addition, the anticancer effect of PPM-18 on other human solid tumor cells was also confirmed by its capacity of reducing cell viability and inhibiting cellular colony formation (Supplementary Figures S1A,B), suggesting that PPM-18 exerts anticancer activity in a broad spectrum of human cancer cells. We next evaluated the anticancer efficiency of PPM-18 through IC_50_ values. As shown in Supplementary Table S1, we found that bladder cancer cells were more sensitive to PPM-18 treatment compared with other human solid tumor cells. To investigate whether PPM-18 has cytotoxic effect on human normal cells, SV-HUC-1 (human bladder immortalized epithelium cells), L02 (human normal liver cells), and HEK293T (human embryonic kidney cells) were employed in this study. As shown in Supplementary Figure S2A, the cell viability of SV-HUC-1, L02, and HEK293T nearly remained intact after exposure to the indicated concentration of PPM-18, implying that PPM-18, at a range of concentration, exerts lower cytotoxicity in human normal cells compared to most cancer cells.

### PPM-18 Triggers Apoptosis in Bladder Cancer Cells

**FIGURE 2:**
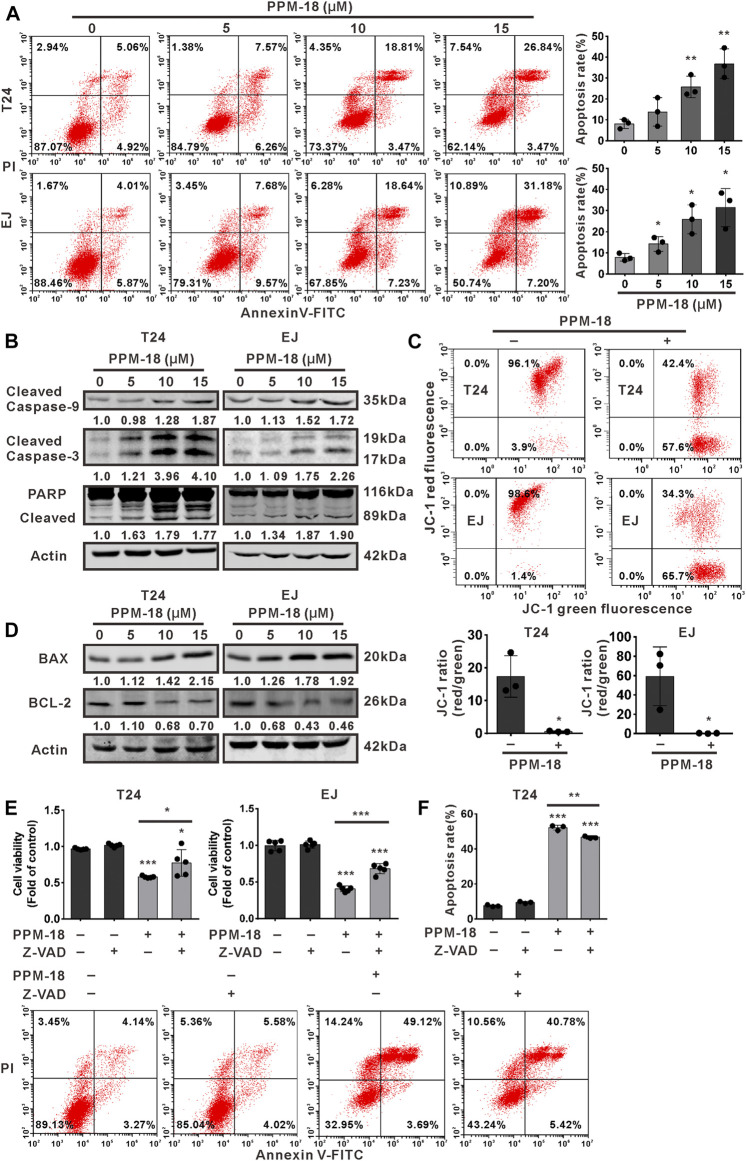
PPM-18 triggers apoptotic cell death in bladder cancer cells. **(A)** T24 and EJ cells were treated with the indicated concentration of PPM-18, and stained with annexin V-FITC and PI. The apoptotic effect of PPM-18 on T24 and EJ cells was measured by flow cytometry. **(B)** Western blotting showed the effect of PPM-18 on the expression of cleaved caspase-9, 3, and PARP in T24 and EJ cells. **(C)** T24 and EJ cells were exposed to 10 μM PPM-18 for 24 h, and stained with JC-1 fluorescent probe. The effect of PPM-18 on the alteration of mitochondria membrane potential was analyzed by flow cytometry. **(D)** Western blotting displayed the effect of PPM-18 on expression of BAX and BCL-2 in T24 and EJ cells. **(E)** and **(F)** The effect of Z-VAD-FMK on PPM-18 reduced the viability of T24 and EJ cells, or PPM-18 triggered apoptosis in T24 cells. Cells treated with 15 μM PPM-18 combined with or without 20 μM Z-VAD-FMK for 24 h, and cell viability and apoptosis were respectively measured by MTS assay and flow cytometry. Data are presented as the mean ± SD of at least three independent experiments. *p < 0.05, **p < 0.01, and ***p < 0.001 vs. the control group, or vs. PPM-18+Z-VAD-FMK.

We next examined the proapoptotic effect of PPM-18 on bladder cancer cells. Flow cytometric analysis showed that PPM-18 significantly increased the apoptosis rate of T24 and EJ cells in a dose-dependent manner (Figure 2A). Furthermore, the cleavage of caspase-9, 3, and PARP, three typical apoptosis indicators, were notably induced in PPM-18–treated T24 and EJ cells (Figure 2B). Additionally, as shown in Figure 2C, the ratio of JC-1 red and green fluorescence that indicates mitochondrial membrane potential was markedly decreased in T24 and EJ cells, upon PPM-18 treatment. We further assessed the impact of PPM-18 on the expression of BAX and BCL-2, two BH3-containing proteins that affect mitochondria membrane potential. Our results showed that PPM-18 stepwise increased BAX expression, whereas it reduced BCL-2 expression in T24 and EJ cells (Figure 2D). These results reveal that PPM-18 is able to trigger mitochondria-associated apoptosis in bladder cancer cells. To investigate whether PPM-18–induced apoptosis in bladder cancer cells is caspase-dependent, Z-VAD-FMK, a pan-caspase inhibitor, was used. Our results showed that treatment with Z-VAD-FMK not only reversed the decrease of cell viability but also reduced apoptosis rate in PPM-18–treated T24 and EJ cells (Figures 2E,F), indicating that PPM-18 induces caspase-dependent apoptosis in bladder cancer cells.

### PPM-18 Induces Autophagy That Facilitates Apoptosis in Bladder Cancer Cells

**FIGURE 3:**
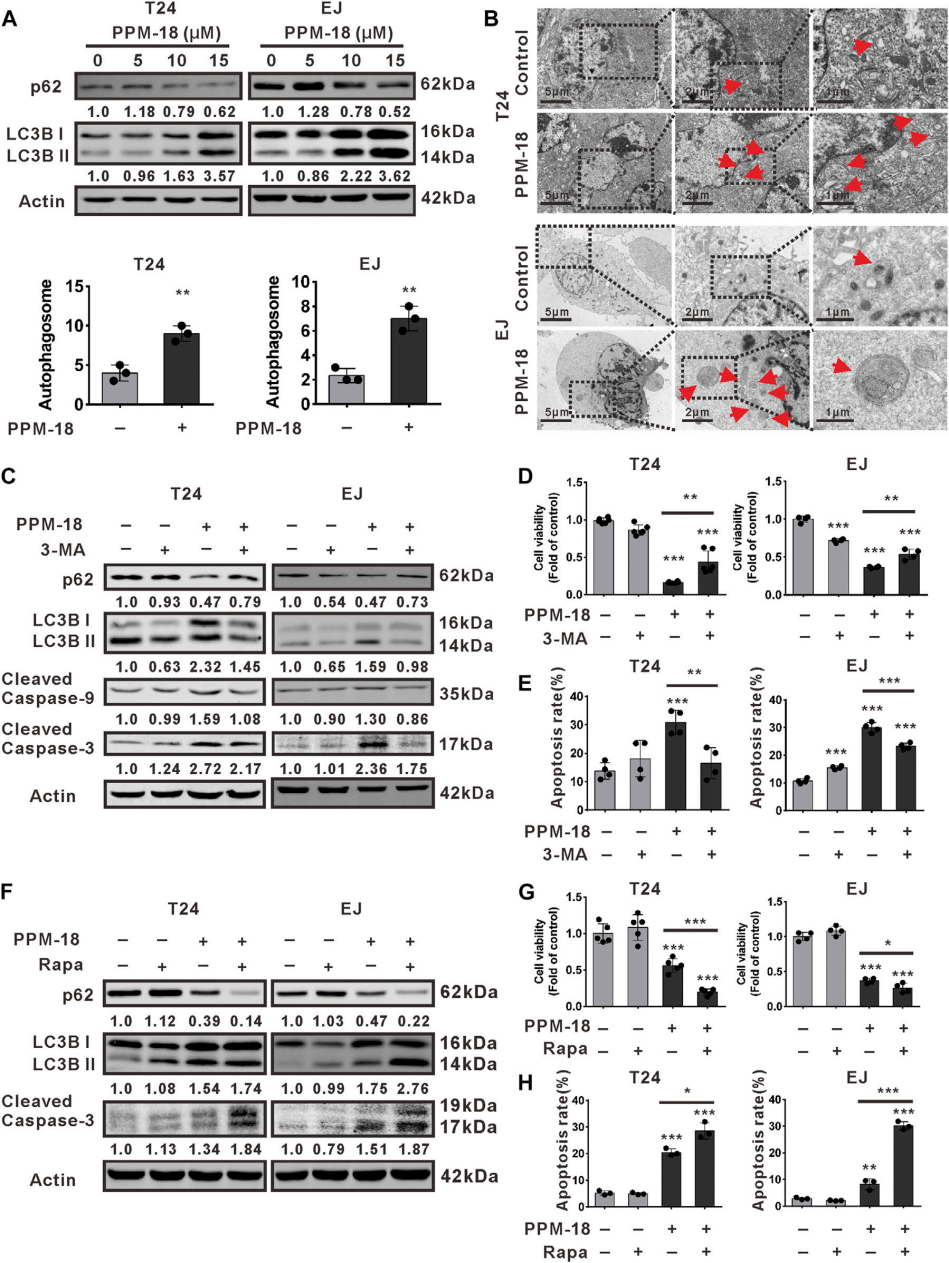
PPM-18 stimulates autophagy that promotes apoptosis in bladder cancer cells. **(A)** T24 and EJ cells were treated with various concentrations of PPM-18 for 24 h. Then cells were harvested for western blotting to detect the expression of p62 and LC3B, two autophagy indicators. **(B)** The formation of autophagosomes in T24 and EJ cells treated with or without 10 μM PPM-18 for 18 h under the transmission electron microscope. Arrows indicated the autophagosomes containing intact and degraded cellular debris. Scale bar: left: 5.0 μm, middle: 2.0 μm, and right: 1.0 μm. **(C)** Western blot showed the effect of 5 mM 3-MA, a typical autophagy inhibitor, on the expression of p62, LC3B, and cleaved caspase-9, 3 in PPM-18–treated T24 and EJ cells. **(D)** and **(E)** T24 and EJ cells were treated with 15 μM PPM-18 combined with or without 5 mM 3-MA, and the effect of 3-MA on PPM-18–reduced viability or PPM-18–triggered apoptosis in T24 cells and EJ cells were measured by MTS assay and flow cytometry, respectively. **(F)** T24 and EJ cells were treated with 15 μM PPM-18 combined with or without 10 μM rapamycin, a typical autophagy agonist, and the effect of rapamycin on PPM-18–induced autophagy and apoptosis were evaluated by western blot. **(G)** The MTS assay demonstrated that rapamycin affected PPM-18–reduced viability in T24 and EJ cells. **(H)** Flow cytometry displayed the effect of rapamycin on PPM-18–triggered apoptosis in T24 and EJ cells. Data are presented as the mean ± SD of at least three independent experiments. **p < 0.01 and ***p < 0.001 vs. the control group, or vs. PPM-18+3-MA. *p < 0.05 and ***p < 0.001 vs. PPM-18+Rapa.

Given that autophagy is another major type of cell death, we next investigated whether PPM-18 could stimulate autophagy in bladder cancer cells. As shown in Figure 3A, PPM-18 dose-dependently decreased the expression of p62, whereas it increased LC3B II expression in T24 and EJ cells. Besides, a considerable number of autophagosomes were detected in T24 and EJ cells following treatment with PPM-18 (Figure 3B). These results indicate that PPM-18 could induce autophagy in bladder cancer cells. To further understand the roles of autophagy in PPM-18–triggered apoptosis, 3-methyladenine (3-MA) and chloroquine (CQ), two typical autophagy inhibitors, and rapamycin, a specific autophagy agonist, were respectively utilized. As expected, treatment with 3-MA or CQ not only repressed PPM-18–induced autophagy but also mitigated PPM-18–triggered apoptosis in T24 and EJ cells (Figures 3C–E, Supplementary Figures S3, S4A). In contrast, both autophagy and apoptosis induced by PPM-18 were further enhanced in T24 and EJ cells, upon rapamycin treatment (Figures 3F–H and Supplementary Figure S4B). These results reveal that PPM-18–induced autophagy could promote apoptosis in bladder cancer cells. To evaluate the autophagic and apoptotic effect of PPM-18 on other human cancer cells, A549, a human lung cancer cell line, and MCF-7, a human breast cancer cell line, were employed. As shown in Supplementary Figures S1C,D, PPM-18 also induced autophagy and apoptosis in A549 and MCF-7 cells, suggesting that PPM-18 could broadly kill human cancer cells. In contrast, no significant autophagy and apoptosis were displayed in L02 cells, a human normal liver cell line, after treatment with the indicated concentration of PPM-18 (Supplementary Figures S2B,C), indicating that PPM-18, at a range of dosage, inefficiently kills human normal cells.

### PPM-18 Induces AMPK-Dependent Autophagy and Apoptosis in Bladder Cancer Cells

**FIGURE 4:**
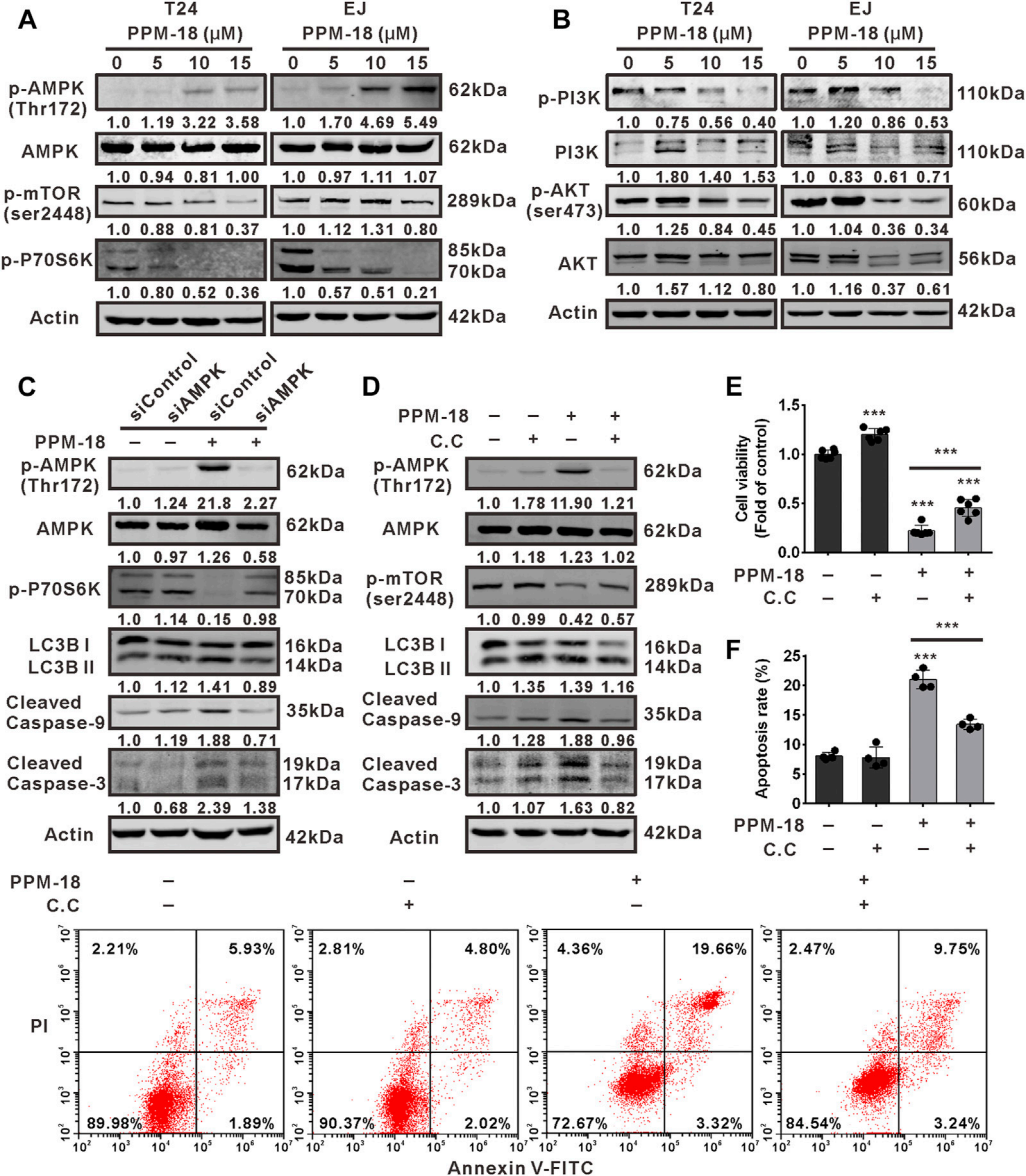
PPM-18 induces AMPK-dependent autophagy and apoptotic cell death in bladder cancer cells. **(A)** and **(B)** T24 and EJ cells were exposed to the indicated concentration of PPM-18 for 24 h, and the expression of phospho-AMPK, AMPK, phospho-mTORC1, phospho-P70S6K, phospho-PI3K, PI3K, phospho-AKT, and AKT were analyzed by western blot. **(C)** and **(D)** Western blot showed the effect of AMPK siRNA or 5 μM Compound C on the phosphorylation of AMPK, mTORC1, and P70S6K, and the expression of LC3B and cleaved caspase-9, 3 in PPM-18–treated T24 cells. **(E)** and **(F)** MTS assay and flow cytometry demonstrated that treatment with Compound C affected PPM-18–reduced viability of T24 cells and PPM-18–induced apoptosis in T24 cells. Data are presented as the mean ± SD of at least three independent experiments. ***p < 0.001 vs. the control group, or vs. PPM-18+Compund C.

To explore the underlying mechanism by which PPM-18 induced autophagy and apoptosis in bladder cancer cells, AMPK/mTORC1 (mammalian target of rapamycin complex 1) pathways that mediate the initiation of autophagy were investigated. As shown in Figure 4A, PPM-18 elevated the phosphorylation of AMPK, whereas it decreased the phosphorylated levels of mTORC1 and P70S6K (a downstream of mTORC1) in T24 and EJ cells, indicating that PPM-18 could activate AMPK and conversely inhibit the mTORC1 pathway in bladder cancer cells. Moreover, the effect of PPM-18 on the activation of PI3K and AKT, the upstream of mTORC1 pathway, was also evaluated in this study. As shown in Figure 4B, the phosphorylated levels of PI3K and AKT were markedly decreased in T24 and EJ cells after exposure to the increasing concentration of PPM-18, implying that PPM-18 could also inhibit PI3K and AKT pathways in bladder cancer cells. To determine whether AMPK activation contributes to PPM-18–induced autophagy and apoptosis in bladder cancer cells, suppression of AMPK was performed. Our results showed that both AMPK siRNA and Compound C, a specific AMPK inhibitor, not only inactivated AMPK but also relieved PPM-18–induced autophagy and apoptosis in T24 cells (Figures 4C–F), indicating that PPM-18 is able to induce autophagy and apoptosis in bladder cancer cells *via* AMPK activation. In addition, the phenomenon that PPM-18 inhibited the mTORC1 pathway was remarkably reversed in T24 cells, upon AMPK repression (Figures 4C,D), suggesting that PPM-18–induced AMPK activation blocks mTORC1 pathway, which leads to autophagy initiation and subsequent apoptosis in bladder cancer cells.

### PPM-18 Increases ROS Production That Contributes to the Decreased Growth of Bladder Cancer Cells

**FIGURE 5:**
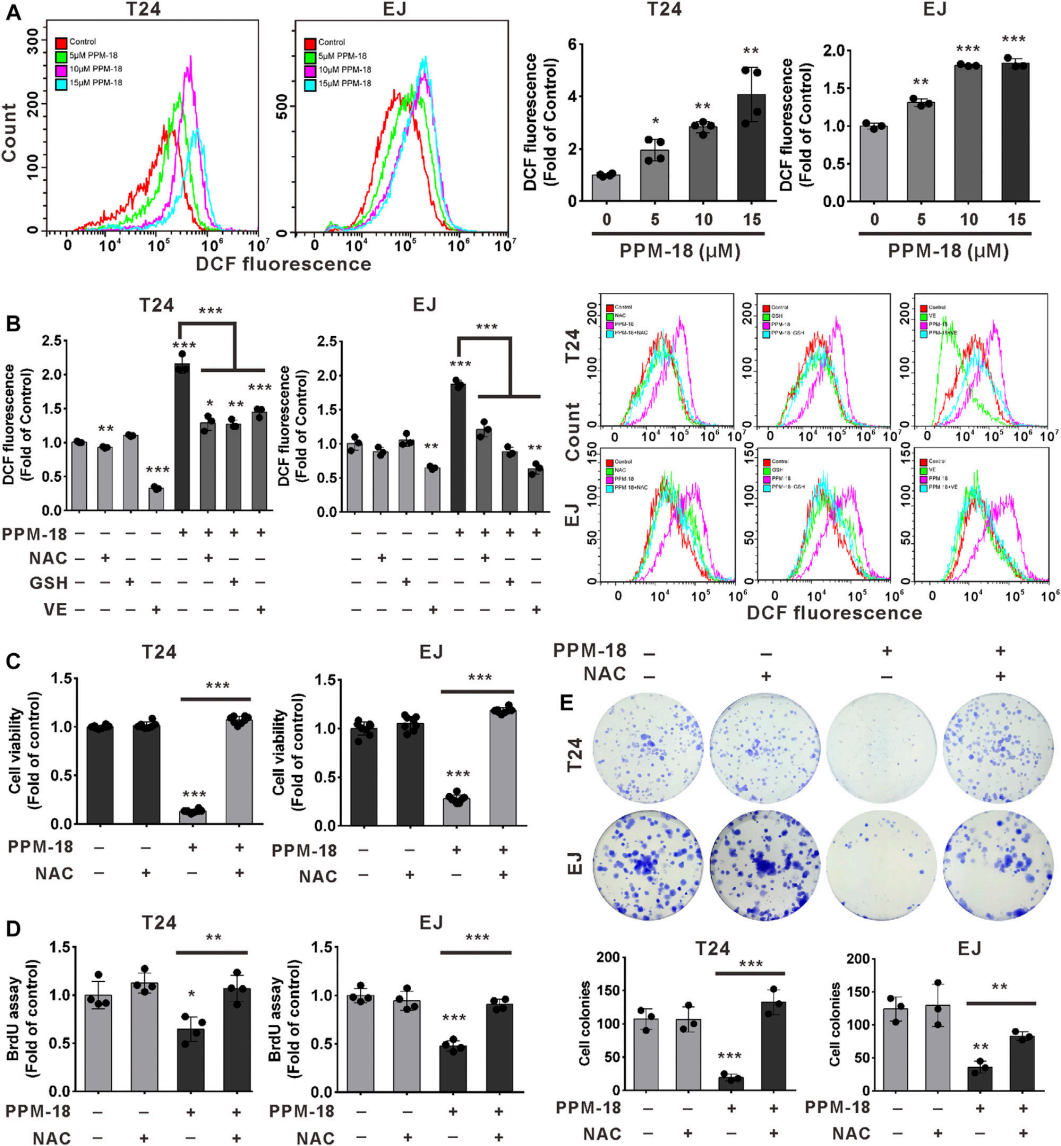
PPM-18–increased ROS generation leads to the inhibition of bladder cancer cell proliferation. **(A)** T24 and EJ cells were dose-dependently treated with PPM-18 for 12 h, and then stained with 1 μM DCFH-DA for 30 min, and intracellular ROS generation was measured by flow cytometry. **(B)** Flow cytometry showed the impact of 6 mM NAC, 5 mM GSH, and 2 mM VE on ROS production in T24 and EJ cells treated with or without 10 μM PPM-18 for 12 h. **(C)** The effect of NAC on the viability of T24 and EJ cells treated with or without PPM-18. **(D)** and **(E)** The effect of NAC on the proliferation of T24 and EJ cells treated with or without PPM-18, using BrdU and cellular colony assays. Data are presented as the mean ± SD of at least three independent experiments. *p < 0.05, **p < 0.01, and ***p < 0.001 vs. the control group. **p < 0.01 and ***p < 0.001 vs. PPM+NAC. ***p < 0.001 vs. PPM+GSH or vs. PPM-18+VE.

Given that oxidative stress is harmful to cancer progression, we next investigated whether PPM-18 could increase ROS production in bladder cancer cells. Our results showed that PPM-18 dramatically upregulated intracellular ROS levels in a dose-dependent manner either in T24 or EJ cells (Figure 5A). Meanwhile, treatment with PPM-18 significantly altered the expression of several antioxidant proteins, such as SOD1 and catalase (Supplementary Figure S5A). To further verify that PPM-18 is capable of increasing ROS generation in bladder cancer cells, N-acetyl-L-cysteine (NAC), glutathione (GSH), and vitamin E (VE)—three ROS scavengers—were utilized. As shown in Figure 5B, PPM-18–elevated ROS production was markedly eliminated in T24 and EJ cells after treatment with NAC, GSH, or VE. Moreover, treatment with NAC significantly attenuated the increased expression of SOD1 and reversed the reduced expression of catalase in PPM-18–treated T24 and EJ cells (Supplementary Figure S5B). These results reveal that PPM-18 indeed increases ROS production in bladder cancer cells. We next investigated whether PPM-18–increased ROS production contributed to the decreased growth of bladder cancer cells. As shown in Figure 5C, PPM-18–reduced viability of T24 and EJ cells was profoundly restored by treatment with NAC. Moreover, NAC treatment also alleviated the inhibitory growth effect of PPM-18 on T24 and EJ cells by BrdU and cellular colony assays (Figures 5D,E). Taken together, our results reveal that PPM-18–increased ROS production is responsible for the decreased proliferation of bladder cancer cells.

### ROS Production Is Required for PPM-18–Induced AMPK-Dependent Autophagy and Apoptosis in Bladder Cancer Cells

**FIGURE 6:**
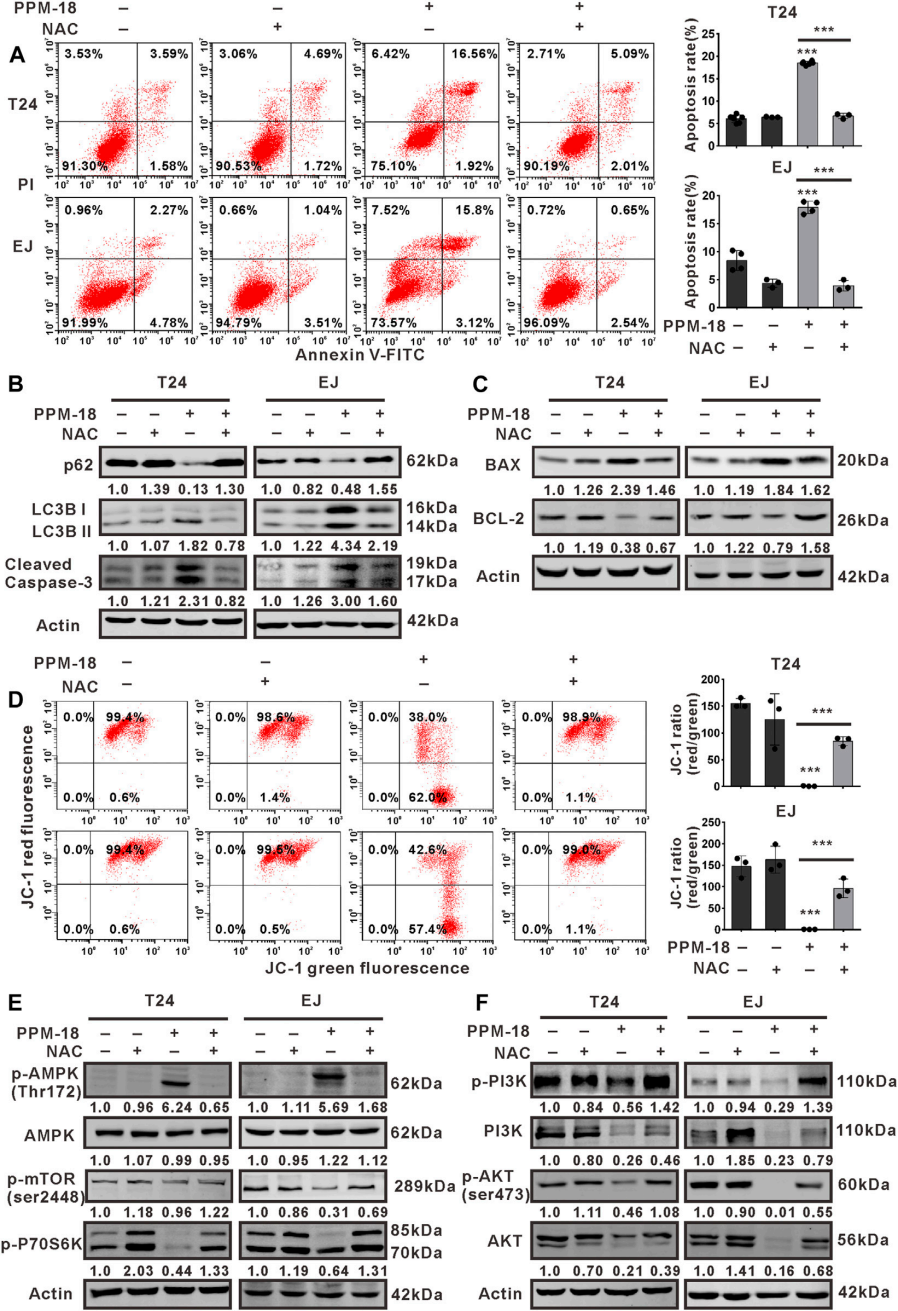
ROS production is required for AMPK-dependent autophagy and apoptosis in PPM-18–treated bladder cancer cells. **(A)** Flow cytometry showed the effect of NAC on PPM-18–triggered apoptosis in T24 and EJ cells. **(B)** and **(C)** Western blot demonstrated that treatment with NAC influenced the expression of p62, LC3B, cleaved caspase-3, BAX, and BCL-2 in T24 and EJ cells treated with or without PPM-18. **(D)** Flow cytometry displayed the effect of NAC on the collapse of mitochondria membrane potential in PPM-18–treated T24 and EJ cells. **(E)** and **(F)** Western blot demonstrated the effect of NAC on AMPK activation and PI3K/AKT/mTORC1 pathways suppression in PPM-18–treated T24 and EJ cells. Data are presented as the mean ± SD of at least three independent experiments. ***p < 0.001 vs. the control group or vs. PPM-18+NAC.

We next investigated whether PPM-18–increased ROS production devoted to autophagy and apoptosis in bladder cancer cells. Our results showed that treatment with NAC, GSH, or VE notably prevented PPM-18–triggered autophagy and apoptosis in T24 and EJ cells (Figures 6A,B and Supplementary Figures S6A,B). Moreover, the collapse of mitochondrial membrane potential triggered by PPM-18 was strikingly restored in T24 and EJ cells, following respective treatment with NAC, GSH, or VE (Figures 6C,D, Supplementary Figure S6C). These results indicate that ROS production is required for PPM-18–induced autophagy and apoptosis in bladder cancer cells. More evidences suggest that ROS can regulate multiple signal pathways that manipulate autophagy and apoptotic cell death. In the present study, the regulatory roles of ROS in AMPK, PI3K/AKT, and mTORC1 pathways were also determined in PPM-18–treated bladder cancer cells. As shown in Figures 6E,F, treatment with NAC remarkably abolished AMPK activation and conversely restored PI3K/AKT and mTORC1 pathways in PPM-18–treated T24 and EJ cells, suggesting that PPM-18–increased ROS production induces AMPK activation and suppresses PI3K/AKT and mTORC1 pathways in bladder cancer cells.

### PPM-18 Exerts Anticancer Activity in Bladder Cancer Xenografts

**FIGURE 7:**
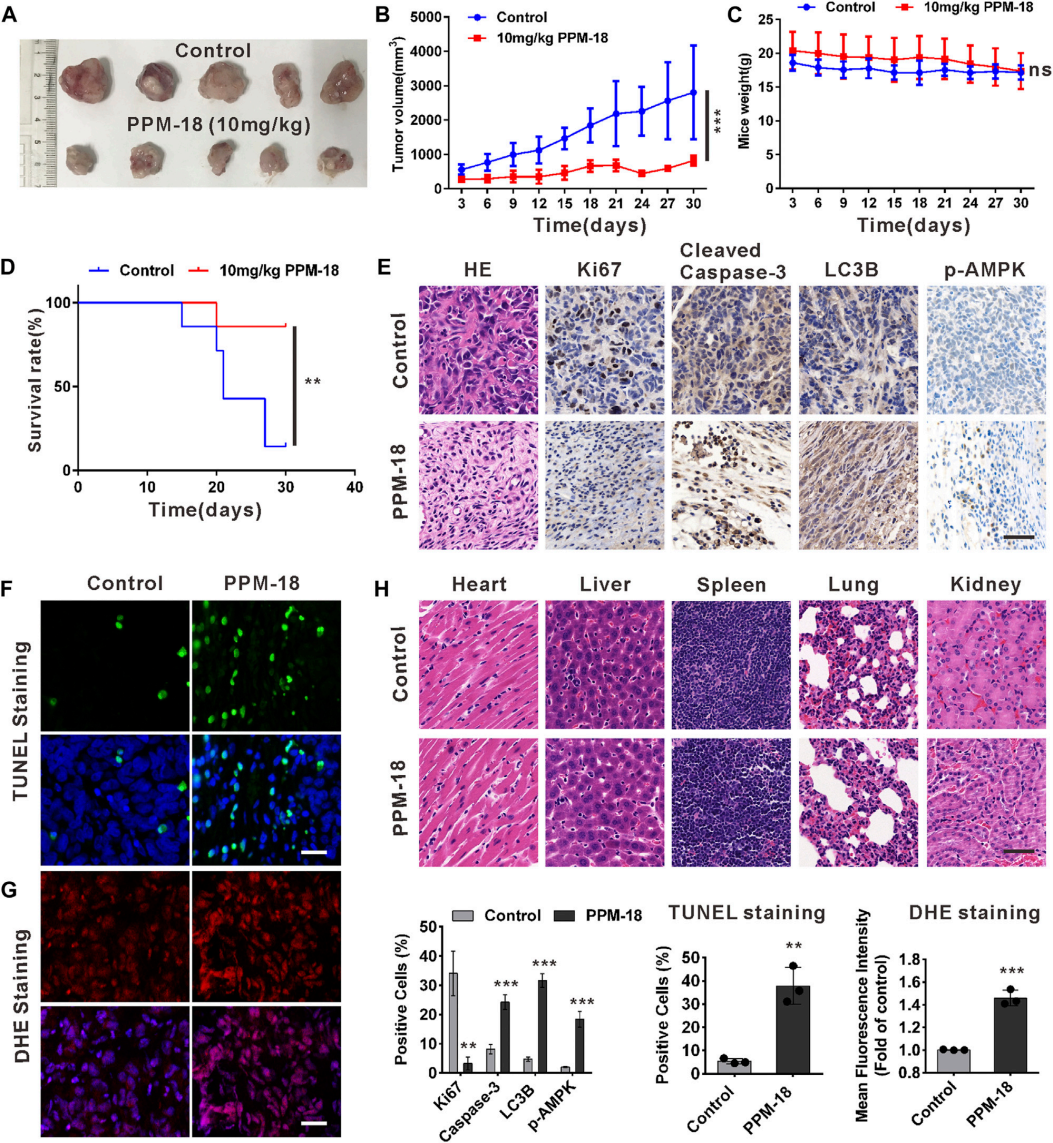
PPM-18 inhibits the growth of bladder cancer xenografts in nude mice. **(A)** and **(B)** Tumor volume changed during administration with or without 10 mg/kg PPM-18 every day for 30 days. **(C)** Mice weight altered during administration with or without PPM-18 for 30 days. **(D)** Survival curves in the two groups of mice. **(E)** The autophagy and apoptosis in bladder tumor tissues from PPM-18–treated group and the control group. HE staining and the expression levels of Ki67, cleaved caspase-3, LC3B, and p-AMPK were examined by immunohistochemistry, scale bar: 50 μm. **(F)** and **(G)** TUNEL staining for apoptosis assay and DHE staining for ROS detecting were analyzed by fluorescence microscope, scale bar: 50 μm. **(H)** The cytotoxic effect of treatment with or without PPM-18 on several important organs from nude mice. HE staining was used to assess the histology, scale bar: 50 μm. Data are presented as the mean ± SD of at least three independent experiments. **p < 0.01 and ***p < 0.001 vs. the control group. ns: no significance.

To determine the anticancer effect of PPM-18 on bladder cancer cells *in vivo*, bladder cancer xenografts were established in nude mice. As shown in Figures 7A,B, administration of PPM-18 at a dose of 10 mg/kg remarkably reduced the tumor sizes, in comparison to the control group. Moreover, treatment with PPM-18 significantly extended the survival (85.7%) of tumor-bearing mice, compared with the lower survival rates (14.3%) in the control group (Figure 7D). To investigate whether PPM-18 induces autophagy and apoptosis in bladder cancer cells *in vivo*, the tumors were excised from the mice and sectioned for immunohistochemistry (IHC) assay. Our results showed that PPM-18 markedly increased the expression of cleaved caspase-3, LC3B, and phospho-AMPK, and reduced the levels of HE staining and Ki67 expression in tumor sections (Figure 7E). Besides, TUNEL and DHE staining assays, respectively, showed the prominent apoptotic cell death and ROS generation in tumor sections from PPM-18–treated mice, compared with that in the control group (Figures 7F,G). These results indicate that PPM-18 is able to induce ROS- and AMPK-related autophagy and apoptosis in bladder cancer cells *in vivo*. On the other hand, the toxic effect of PPM-18 on mice was evaluated as well. During PPM-18 administration, no significant discrepancy of mice weight was displayed between the control and PPM-18–treated group (Figure 7C). Furthermore, no major organ-related toxicities were exhibited in PPM-18–treated mice (Figure 7H). Taken together, our data suggest that PPM-18 could exert anticancer activity in bladder tumors *in vivo* with low toxicity.

## Discussion

To date, the outstanding anticancer functions of vitamin K as well as its analogs have been revealed in many studies (Dasari et al., 2017; Xv et al., 2018). Both vitamin K and its analogs were proved to exhibit their excellent abilities against the growth of multiple cancer cells through arresting cell cycle, regulating cell differentiation, and inducing autophagic or apoptotic cell death (Karasawa et al., 2013; Maniwa et al., 2015; Vera et al., 2019). However, the functions of PPM-18, as a novel analog of vitamin K, against human solid tumor were rarely reported. In this study, the anticancer effect of PPM-18 on bladder cancer cells was demonstrated. Our results showed that PPM-18 exerted inhibitory growth effect on bladder cancer cells through reducing cell viability, inhibiting BrdU incorporation, and hampering cellular colony formation. Amazingly, similar results were also obtained in other PPM-18–treated human cancer cells. These results indicate that PPM-18, like vitamin K, is endowed with remarkable anticancer features in a wide range of cancer cells. Apart from antagonistic roles in cancer cell proliferation, PPM-18, in our study, was also proved to induce autophagy and apoptosis in bladder cancer cells. Autophagy has been suggested to play controversial roles in cancer therapy. In some cases, autophagy is confirmed to have cytoprotective effects on the survival of cancer cells, and autophagy inhibition could potentiate the sensitivity of cancer cells to anticancer drugs, such as cisplatin and doxorubicin (Li et al., 2017; Xu et al., 2017; Tian et al., 2019). However, more evidences reveal that autophagy activation could promote cancer cell death (Denton and Kumar, 2019; Kriel and Loos, 2019). In this study, PPM-18–triggered apoptosis was markedly suppressed in bladder cancer cells following autophagy inhibition. Conversely, the apoptotic effect of PPM-18 on bladder cancer cells was significantly enhanced, upon autophagy activation. These results suggest that PPM-18 is able to induce autophagy, which promotes the subsequent apoptosis in bladder cancer cells.

AMPK, as a metabolic regulator, has recognized to be a tumor suppressor, due to its involvement in multiple networks, including LKB1, TSC1-TSC2 complex, and P53 (Thirupathi and Chang, 2019; Ma et al., 2020). Consequently, it is no doubt that deficiency of AMPK is prevalent in some types of cancer cells. Recently, AMPK activators have made massive progress in cancer treatment by restoring AMPK activity (Li et al., 2019; Liu et al., 2019; Si et al., 2019). In our study, PPM-18 remarkably elevated the phosphorylation of AMPK in T24 and EJ cells, suggesting that PPM-18 is capable of promoting AMPK activation in bladder cancer cells. Furthermore, the autophagic and apoptotic effects of PPM-18 on T24 cells were profoundly mitigated upon AMPK suppression, implying that PPM-18 induces autophagy and apoptosis through AMPK activation in bladder cancer cells. As suggested from latest studies, AMPK is able to inhibit the mTORC1 pathway by phosphorylating raptor, a subunit of mTORC1, or phosphorylating TSC2, the upstream regulator of mTORC1 (Herzig and Shaw, 2018), which caused the initiation of autophagy. In line with these studies, our results showed that PPM-18 markedly repressed the mTORC1 pathway in bladder cancer cells, along with AMPK activation and autophagy induction. Importantly, abrogating AMPK activation not only suppressed PPM-18–induced autophagy but also restored the mTORC1 pathway in bladder cancer cells. These results indicate that PPM-18–activated AMPK could suppress the mTORC1 pathway, which further results in the occurrence of autophagy in bladder cancer cells.

Recent studies have suggested that cancer cells prevalently exhibit much higher ROS levels than normal cells, due to mitochondria dysfunction, oncogene activation, and antioxidant imbalance (Panieri and Santoro, 2016). Consequently, further increasing ROS generation that leads to oxidative stress–induced cell death has long been regarded as the exact anticancer mechanism for many drugs (Conklin, 2004). In our studies, the prooxidant role of PPM-18 in bladder cancer cells was also determined. Our results clearly showed that PPM-18 remarkably promoted ROS production in bladder cancer cells, in accompany with the altered expression of antioxidant proteins, such as SOD1 and catalase. Moreover, blocking ROS generation not only prevented autophagy but also rescued bladder cancer cells from PPM-18–triggered apoptotic cell death. These results suggest that PPM-18 could increase ROS generation that results in oxidative stress–induced autophagy and apoptosis in bladder cancer cell. Growing evidences indicate that AMPK activation also depends on ROS regulation, which contributes to the subsequent autophagy and apoptosis in cancer cells (Rabinovitch et al., 2017; Choi, 2018). Concordantly, our data showed that PPM-18–induced AMPK activation was strikingly abrogated following ROS elimination, implying that ROS act as an upstream of AMPK and induce AMPK activation in PPM-18–treated bladder cancer cells. Many previous studies suggest that PI3K/AKT and mTORC1 pathways are commonly suppressed in response to oxidative stress, which promotes autophagic or apoptotic cell death (Mi et al., 2016; Song et al., 2017; Kim et al., 2019). In the present study, the phenomenon that PPM-18–reduced the phosphorylation of PI3K/AKT and mTORC1 was remarkably compromised, upon ROS elimination, implying that PPM-18–increased ROS production blunts PI3K/AKT and mTORC1 pathways, which ultimately results in autophagy and apoptosis in bladder cancer cells.

**FIGURE 8:**
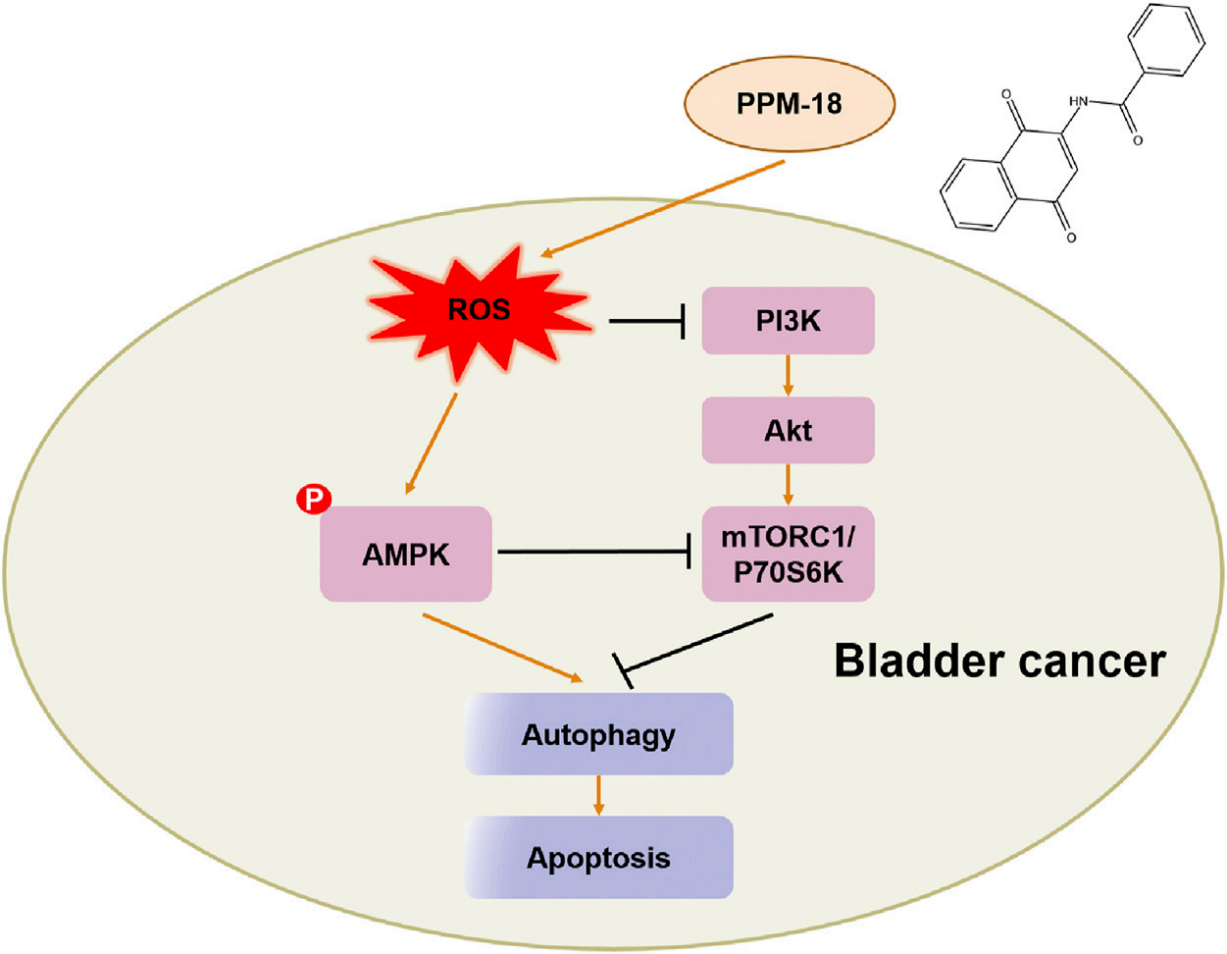
Schematic diagram of signaling pathways associated with PPM-18–induced autophagy and apoptosis in bladder cancer cells.

In conclusion, the anticancer effect of PPM-18 on bladder cancer cells *in vitro* and *in vivo* had been exhibited in our study. Furthermore, the detailed mechanism of PPM-18 against bladder cancer cells was also demonstrated. In our study, PPM-18 was proved to increase ROS production that caused oxidative stress in bladder cancer cells. In response to oxidative stress, AMPK was activated, and PI3K/AKT and mTORC1 pathways were suppressed in turn, which leads to AMPK-dependent autophagy and apoptosis in bladder cancer cells (Figure 8). Given the excellent anticancer abilities of PPM-18 in bladder cancer cells, without significant cytotoxic effect on normal cells *in vitro* and *in vivo*, our study might provide a good strategy for future bladder cancer treatment.

## Data Availability

The original contributions presented in the study are included in the article/Supplementary Material; further inquiries can be directed to the corresponding authors.
