# Supplementary material for: PPM-18, an Analog of Vitamin K, Induces Autophagy and Apoptosis in Bladder Cancer Cells Through ROS and AMPK Signaling Pathways
Source: Front Pharmacol. 2021 Jul 9;12:684915. doi: 10.3389/fphar.2021.684915 (PMC8299005; doi:10.3389/fphar.2021.684915)
Supplement: Supplementary file 1 [file DataSheet1.docx]

Supplementary Material


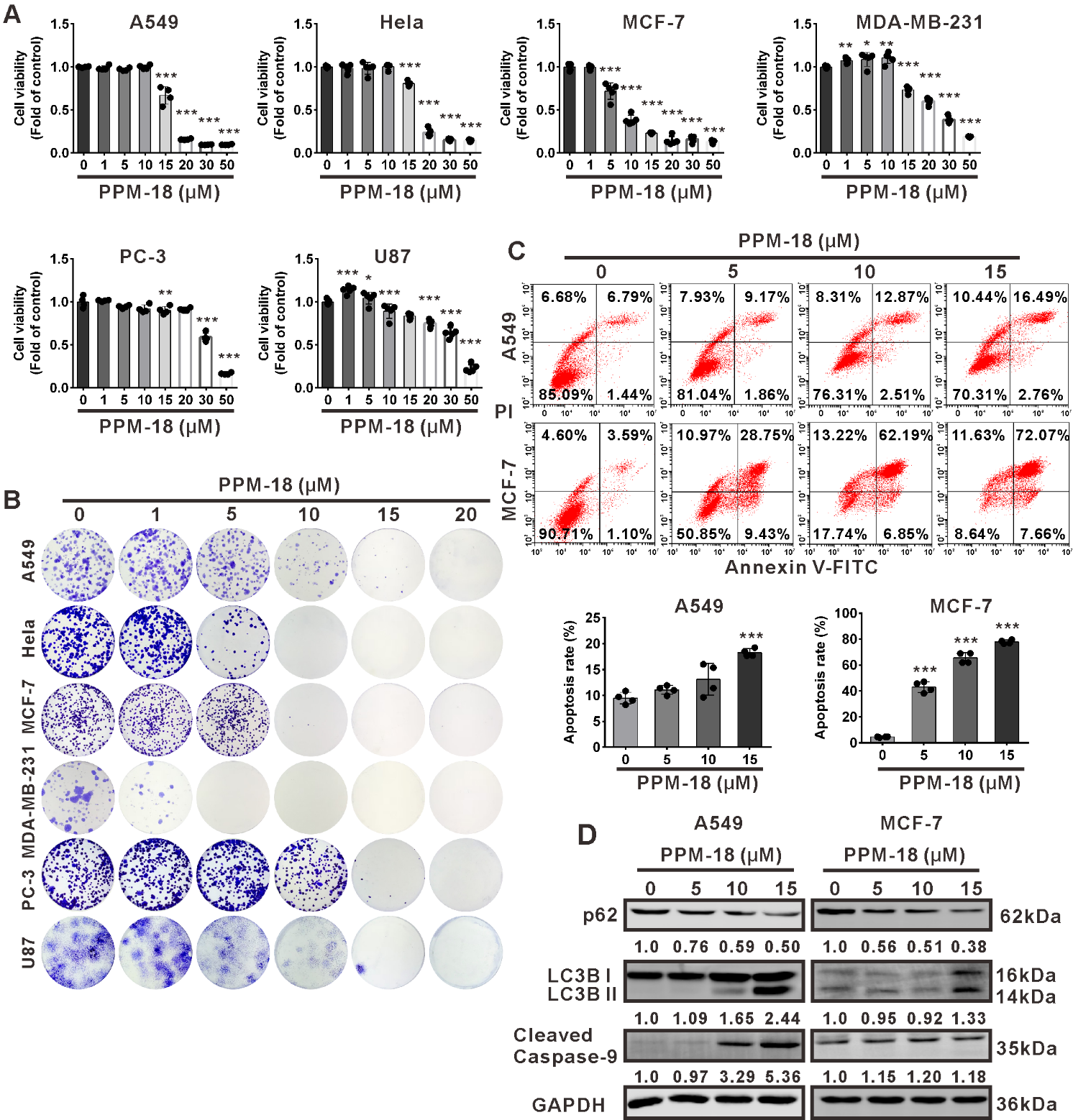


**Supplementary Figure 1.** PPM-18 inhibits the proliferation, and induces autophagy and apoptosis in other human cancer cell lines *in vitro*. **(A)** The cytotoxic effect of PPM-18 on other human cancer cell lines. Human lung cancer A549, human cervical cancer Hela, human breast cancer MCF-7 and MDA-MB-231, human prostate cancer PC-3, and human glioma U87 cells were incubated with increasing doses of PPM-18 (0‑50 µM) for 24 h to analyze cell viability using MTS assay. **(B)** PPM-18 affected the colony formation of other human cancer cells. Different cancer cells were incubated with PPM-18 at 0-20 µM for 10 days. Representative colony images are presented. **(C)** Flow cytometry showed the effect of PPM-18 on the apoptosis rate of A549 and MCF-7 cells. **(D)** Western blot displayed the effect of PPM-18 on the expression of p62, LC3B and cleaved caspase-9 in A549 and MCF-7 cells. Data are presented as the mean ± SD of at least three independent experiments. **P* < 0.05, ** *P*<0.01 and ****P* < 0.001 vs. the control group.


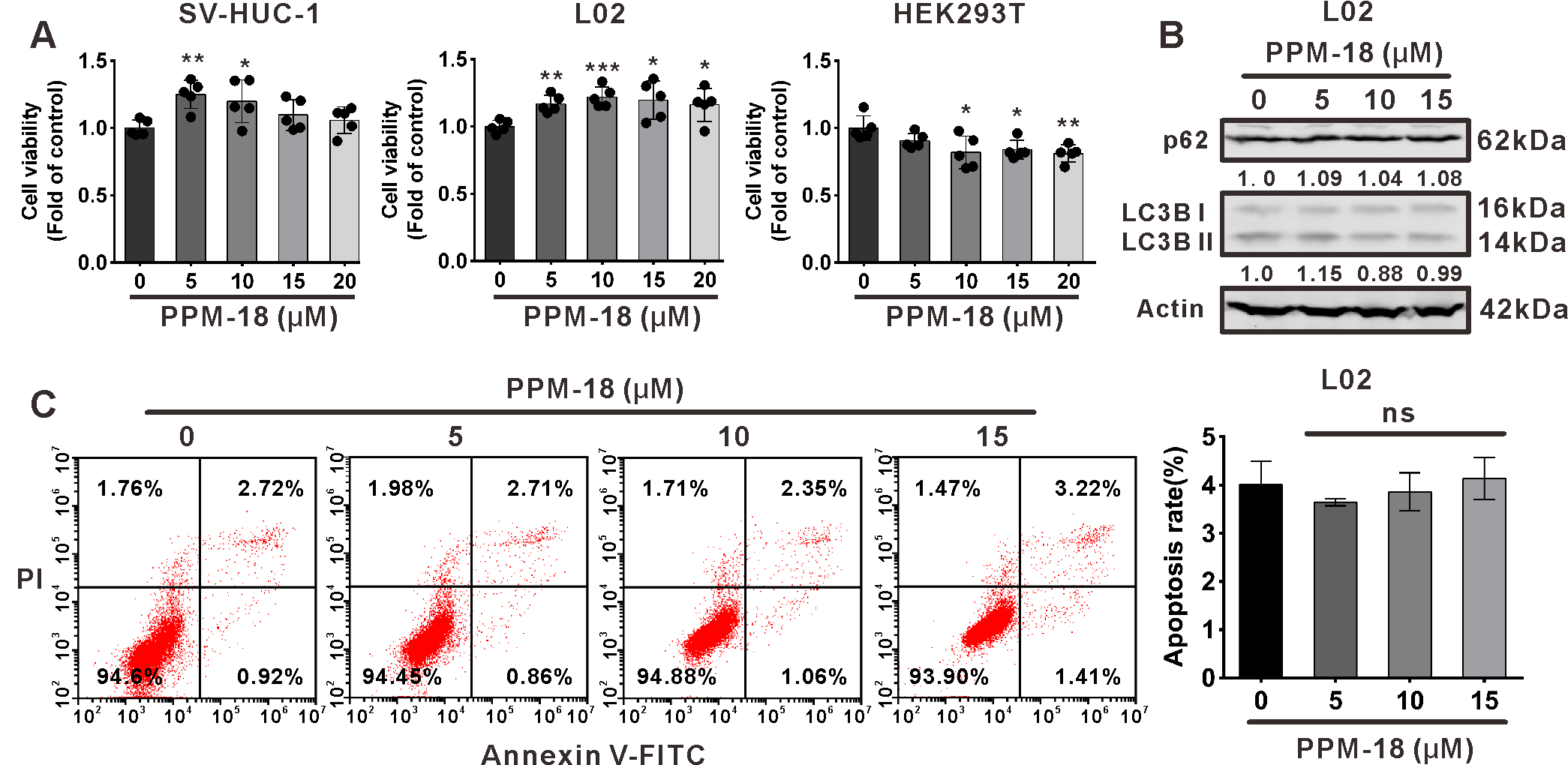


**Supplementary Figure 2.**  PPM-18 exhibits low cytotoxity in human normal cells. Cells were treated with various concentrations of PPM-18 for 24 h. **(A)** MTS assay exhibited the effect of PPM-18 on the viability of SV-HUC-1, L02 and HEK293T cells. **(B)** Western blot showed the effect of PPM-18 on the expression of p62 and LC3B in L02 cells. **(C)** Flow cytometry displayed the effect of PPM-18 on the apoptosis rate in L02 cells. Data are presented as the mean ± SD of at least three independent experiments. **P* < 0.05, ** *P*<0.01 and ****P* < 0.001 vs. the control group. ns: no significance.


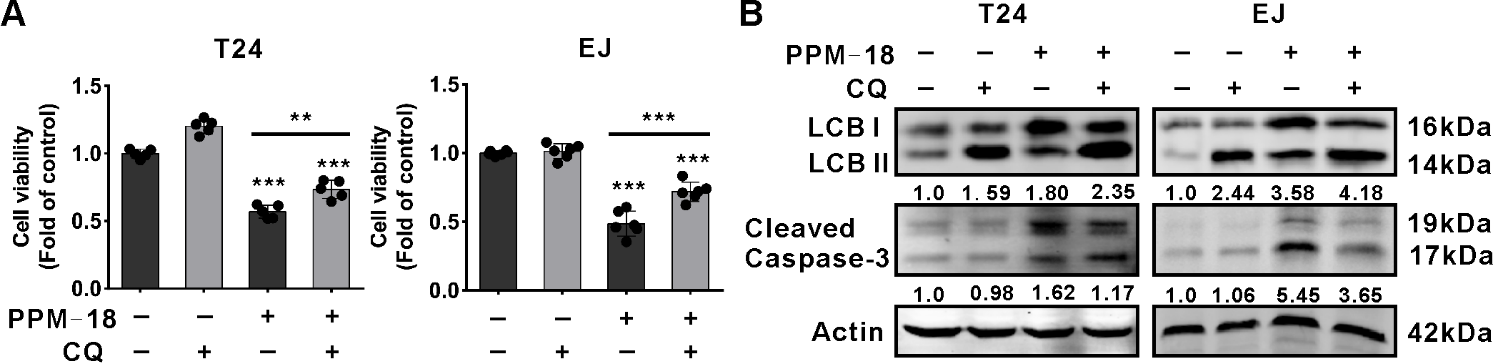


**Supplementary Figure 3.** Treatment with chloroquine (CQ) attenuates PPM-18-induced autophagy and apoptosis in bladder cancer cells. **(A)** MTS assay showed the effect of 10 μM CQ on the viability of T24 and EJ cells treated with or without 15μM PPM-18. **(B)** Western blot demonstrated that CQ affected the expression of LC3B and cleaved caspase-3 in T24 and EJ cells treated with or without PPM-18. ****P* < 0.001 vs. the control group or vs. PPM-18+CQ. ** *P*<0.01 vs. PPM-18+CQ.


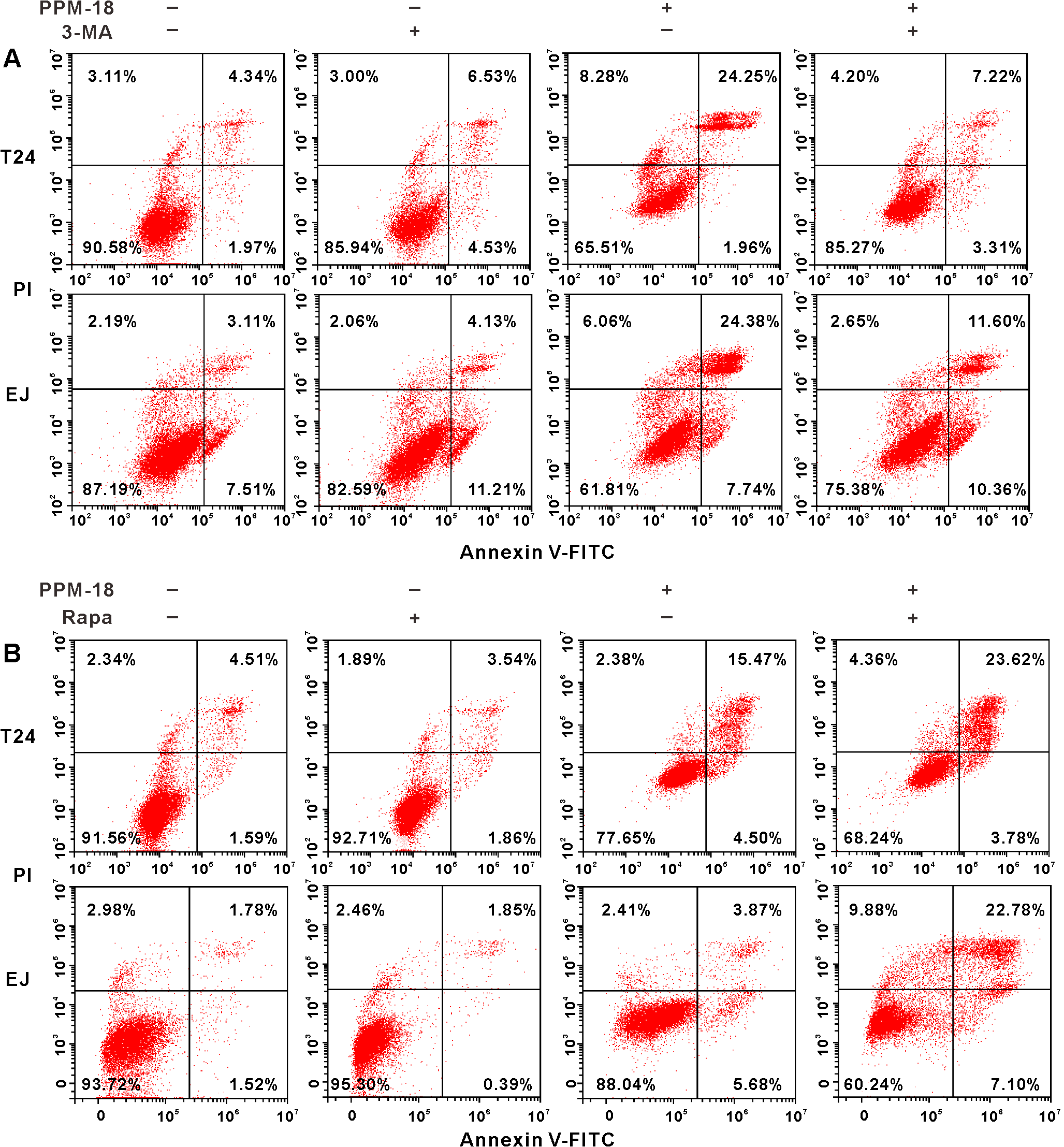


**Supplementary Figure 4.** The scatter diagrams of apoptosis detection by flow cytometry in T24 and EJ cells treated with 5 mM 3-MA **(A)** or 10 μM rapamycin **(B)** combined with or without 15 μM PPM-18 for 24 h.


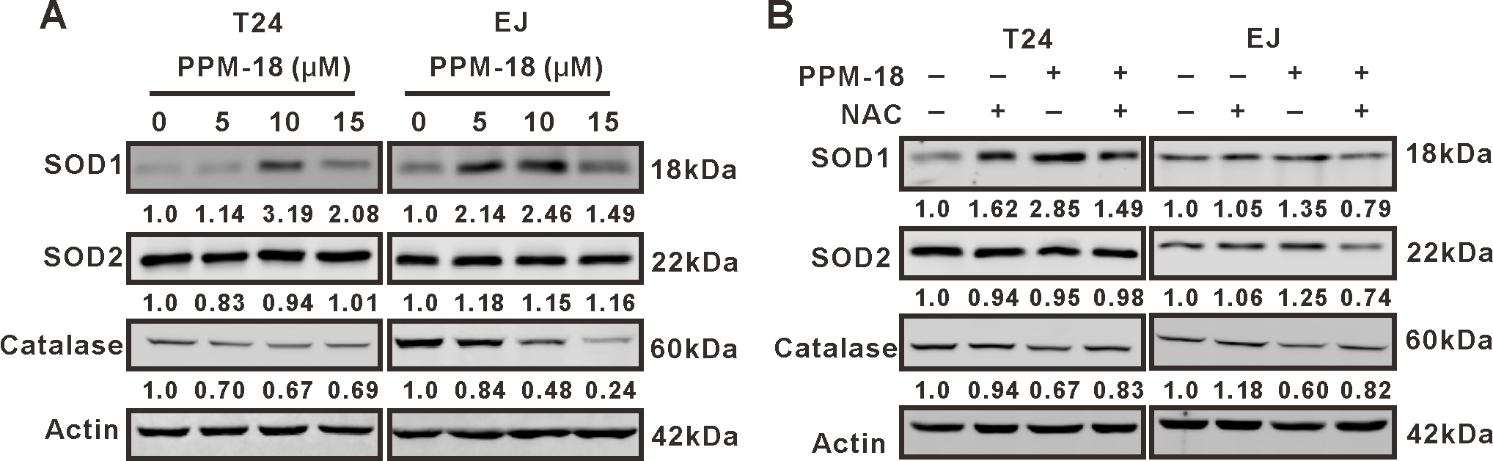


**Supplementary Figure 5.** PPM-18 alters the expression of several antioxidant proteins in bladder cancer cells. **(A)** Western blot showed the effect of PPM-18 on the expression of SOD1, SOD2 and catalase in T24 and EJ cells. **(B)** NAC treatment affected the expression of SOD1, SOD2 and catalase in T24 and EJ cells treated with or without PPM-18.

**
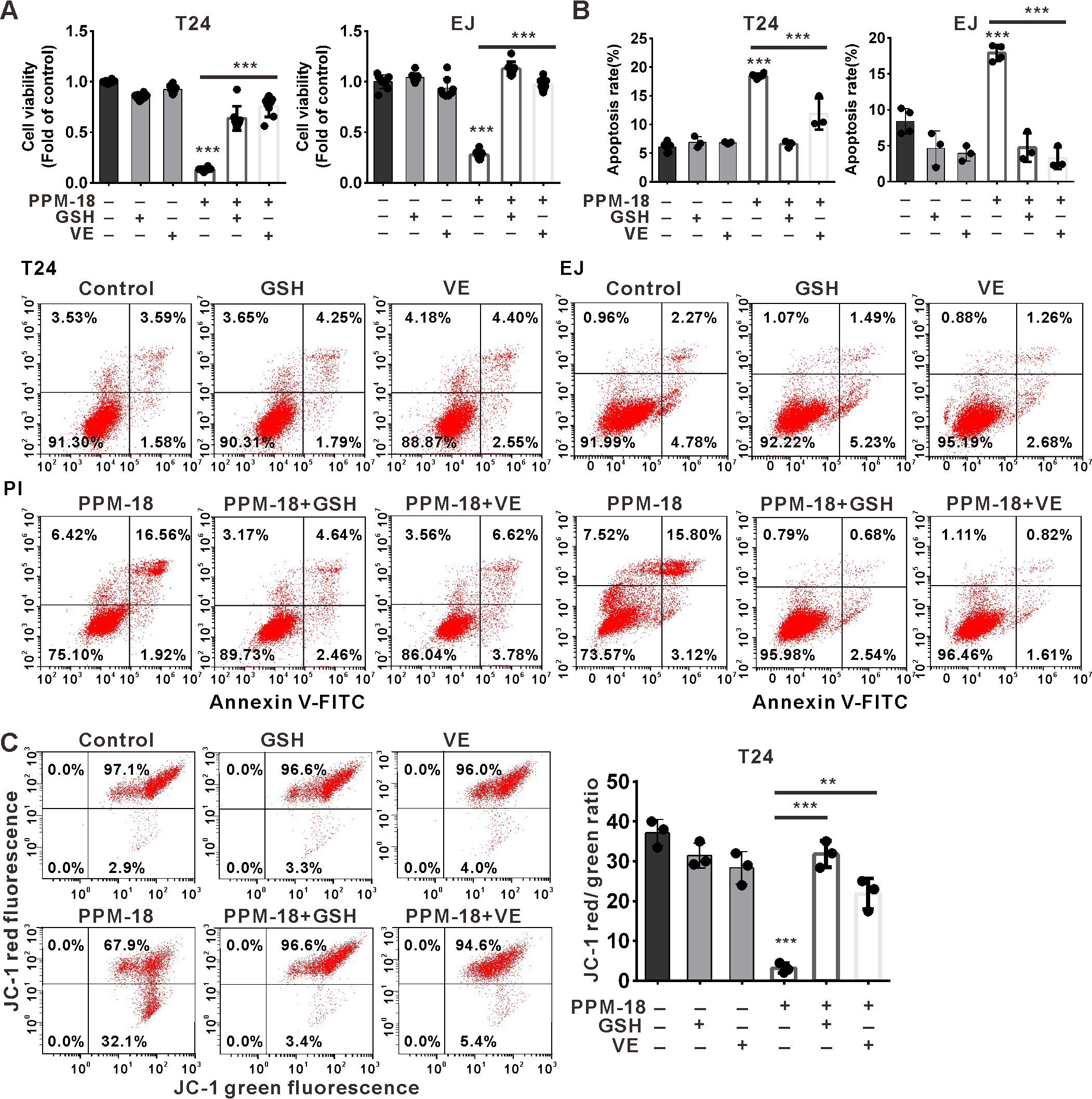
**

**Supplementary Figure 6.**  Effect of reactive oxygen species scavengers on the viability and apoptosis in PPM-18-treated bladder cancer cells. T24 and EJ cells were treated by 10 µM PPM-18 combined with or without GSH (5 mM) and VE (2 mM) for 24 h. **(A)** Cell viability was evaluated by MTS assay. **(B)** Cell apoptosis was performed by flow cytometry. **(C)** Mitochondrial membrane potential was measured by flow cytometry. Data are presented as the mean ± SD of at least three independent experiments. ****P* < 0.001 vs. the control group or vs. PPM-18+GSH. ** *P*<0.01 and ****P* < 0.001 vs. PPM-18+VE.

**Supplementary TABLE 1** IC_50_ of PPM-18 against various human cancer cell lines for 24 h.

| **Cell lines** | **IC_50_(μM)** |
| --- | --- |
| **T24** | 13.17±0.82 |
| **EJ** | 12.21±1.02 |
| **A549** | 15.52±0.73 |
| **Hela** | 17.62±0.23 |
| **MCF-7** | 7.82±0.21 |
| **MDA-MB-231** | 21.30±1.52 |
| **PC-3** | 33.13±0.28 |
| **U87** | 34.01±1.24 |


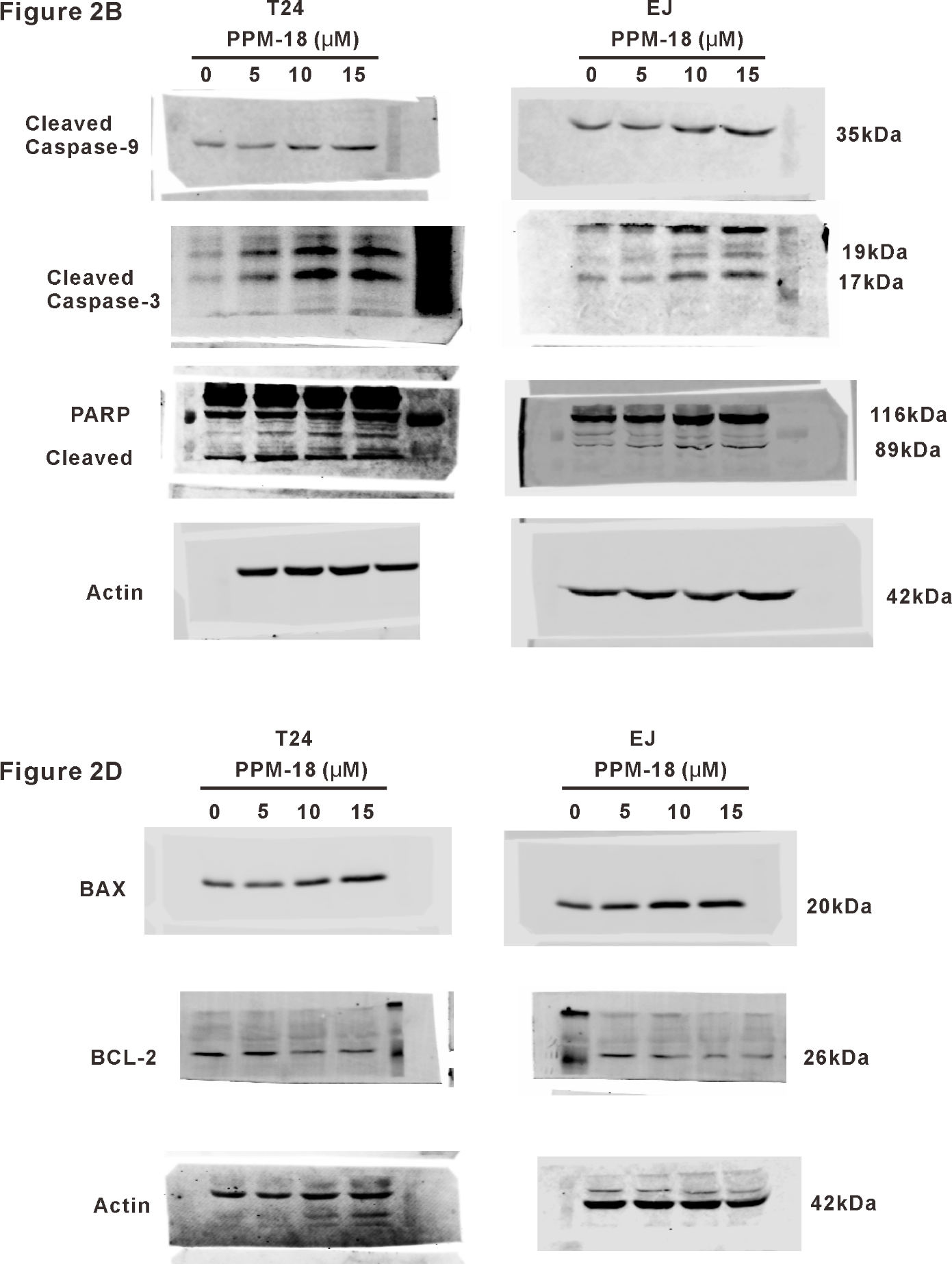


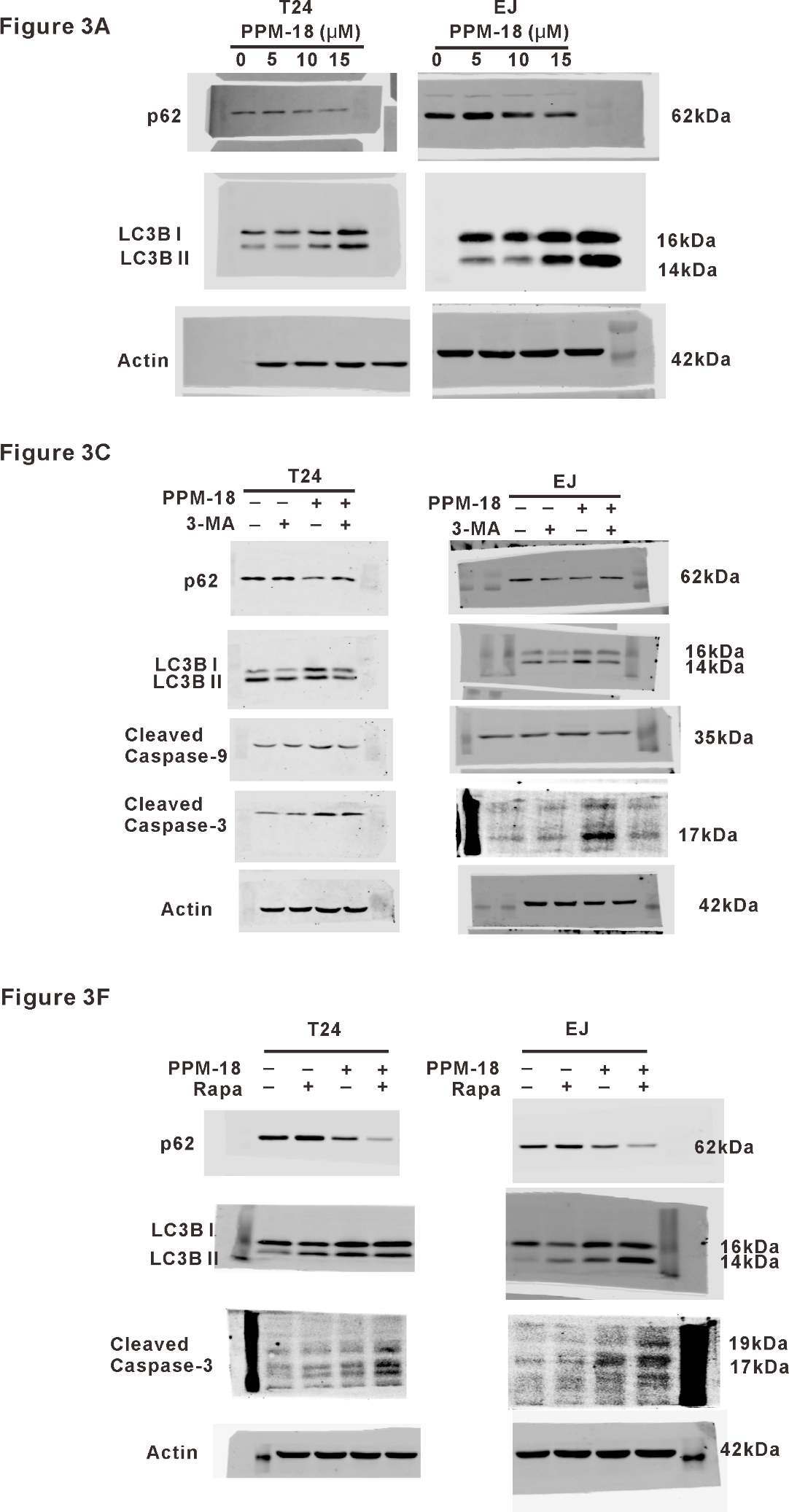


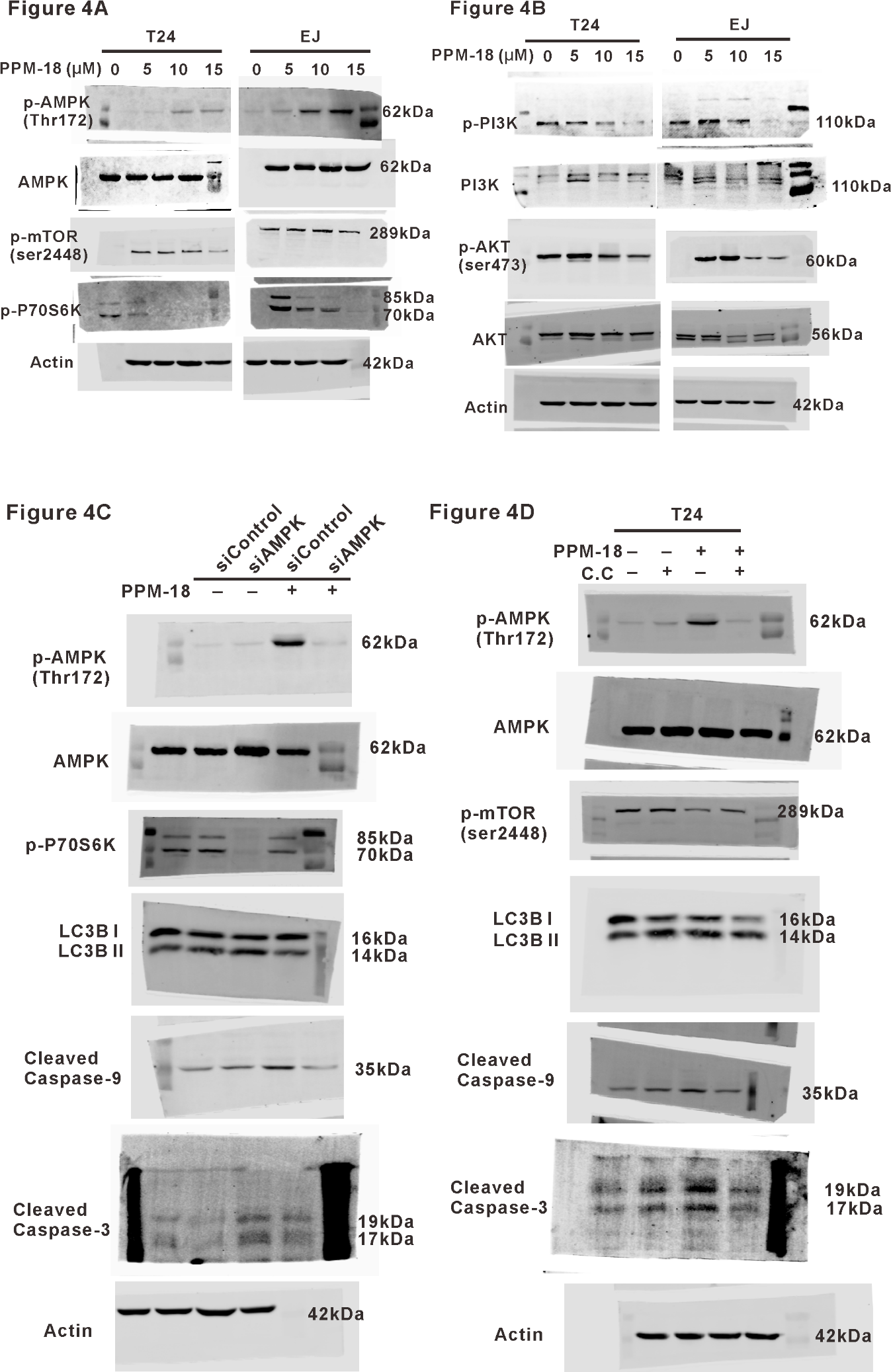


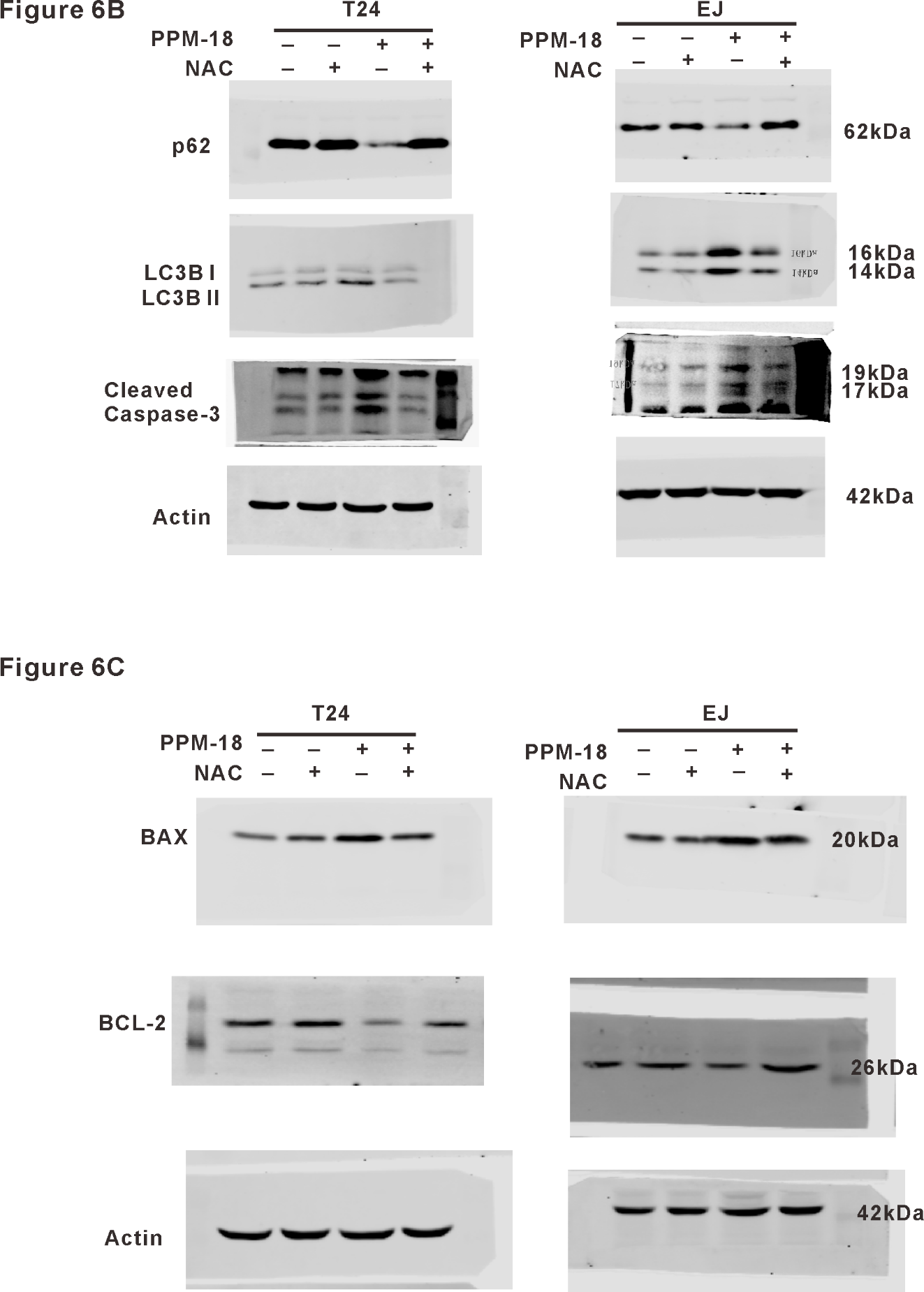


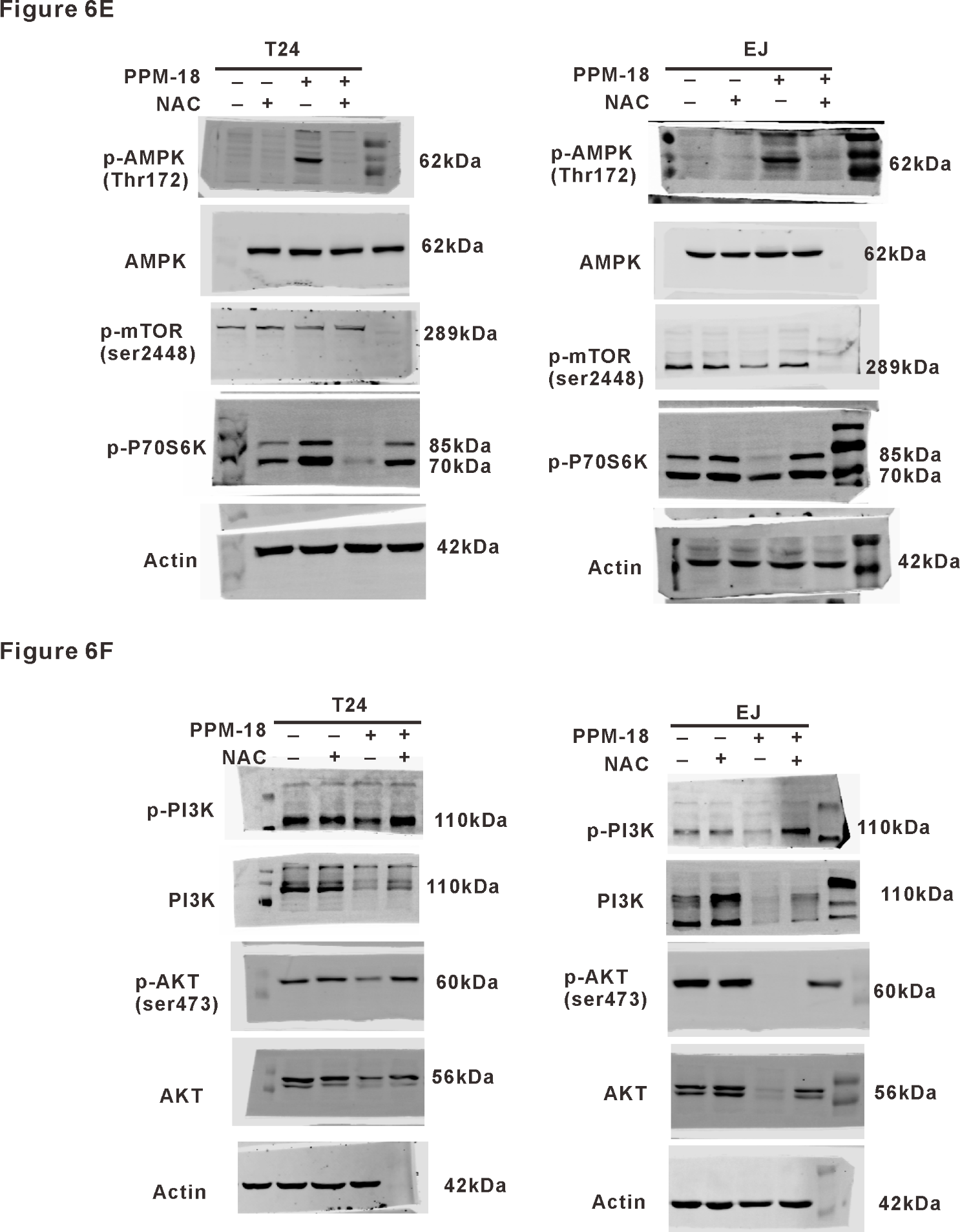


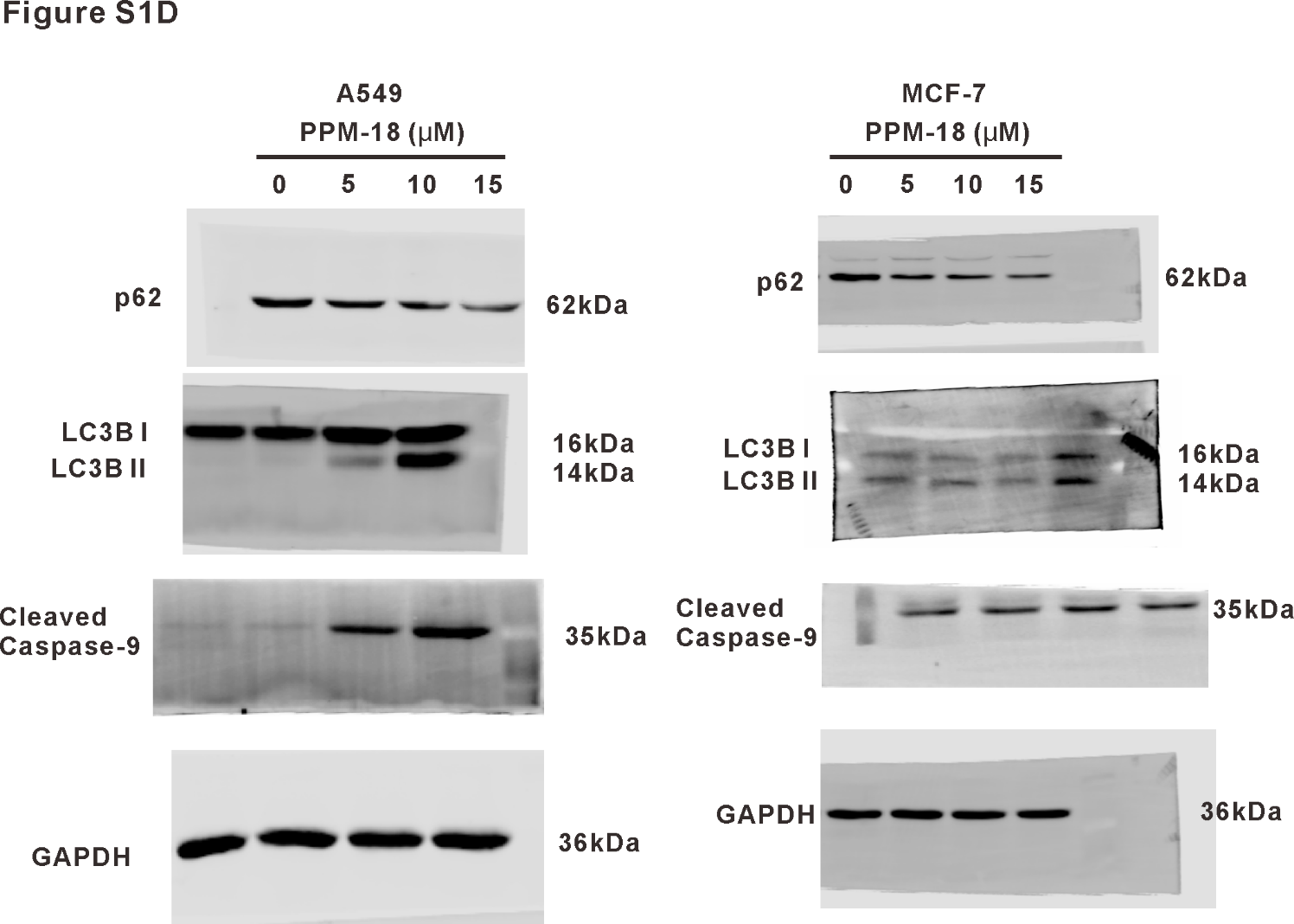


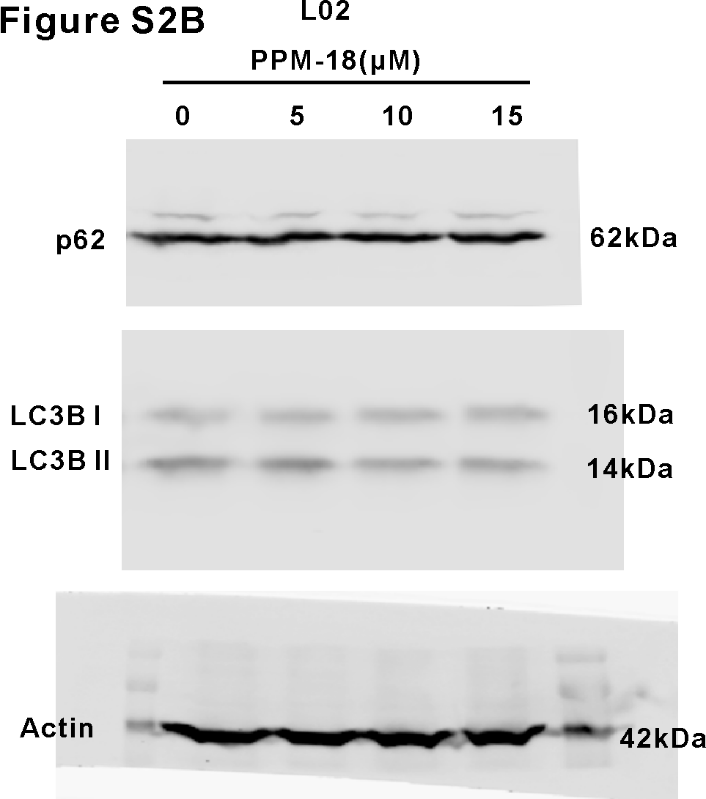


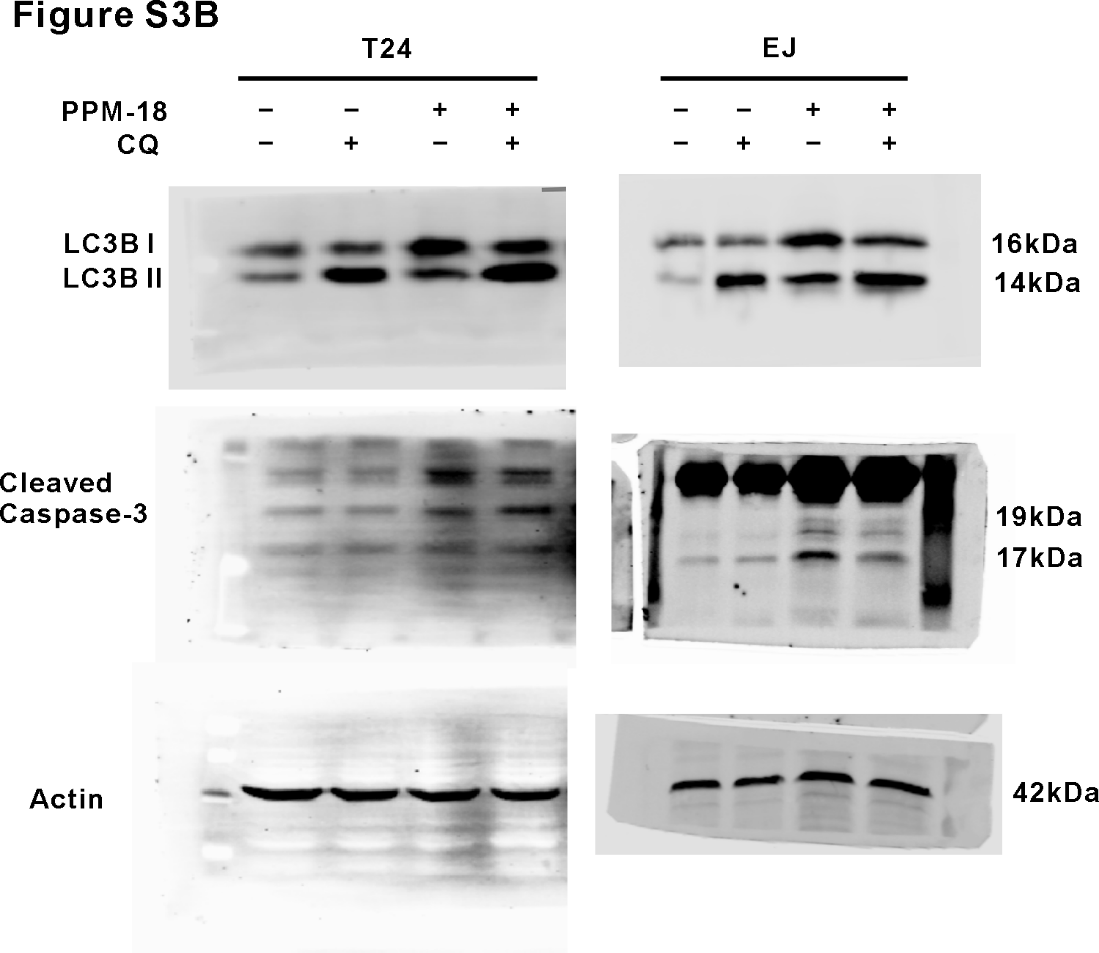


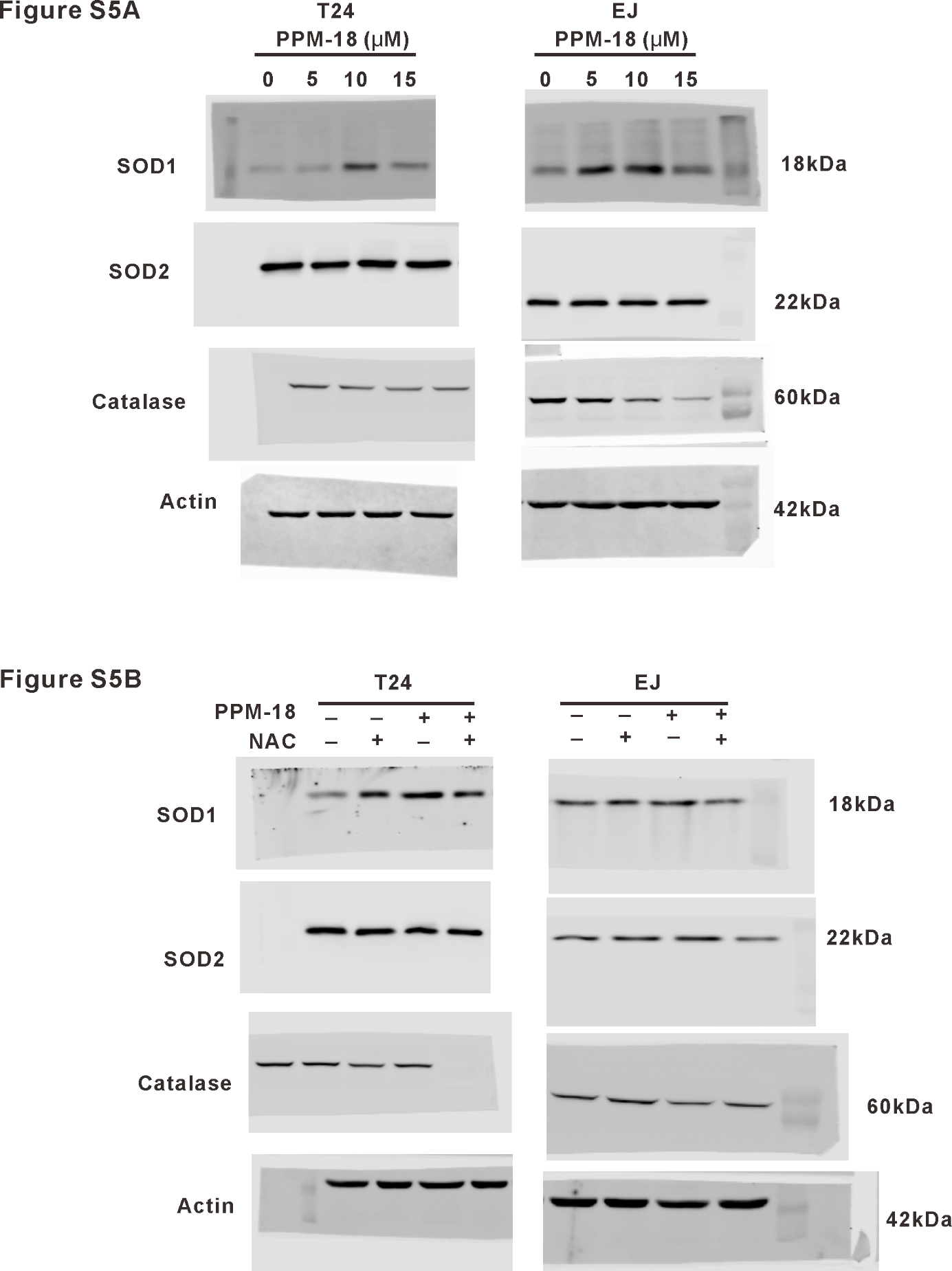
Uncropped western blot bands
